# A novel tumor-associated neutrophil gene signature for predicting prognosis, tumor immune microenvironment, and therapeutic response in breast cancer

**DOI:** 10.1038/s41598-024-55513-8

**Published:** 2024-03-04

**Authors:** Jianyou Zhang, Xinbo Wang, Zhonglai Zhang, Fuyi Ma, Feng Wang

**Affiliations:** 1 Department of Breast Disease, Weifang People’s Hospital, Weifang, No.151, Guangwen Street, Kuiwen District, Shandong China; 2 Department of General Surgery, Gaomi People’s Hospital, Weifang, Shandong China

**Keywords:** Tumor-associated neutrophil, Breast cancer, Triple-negative breast cancer, Drug sensitivity, Prognostic signature, Immune microenvironment, Cancer, Breast cancer, Cancer genetics, Cancer microenvironment, Cancer models

## Abstract

Tumor-associated neutrophils (TANs) can promote tumor progression. This study aimed to investigate the molecular signature that predict the prognosis and immune response of breast cancer (BRCA) based on TAN-related gene (TANRG) expression data. The RNA-seq data of BRCA were gathered from The Cancer Genome Atlas (TCGA) and gene expression omnibus (GEO) datasets. Univariate Cox regression analysis and the least absolute shrinkage and selection operator for selecting prognostic genes. A neo-TAN-related risk signature was constructed by multivariate Cox regression analysis. Time-dependent receiver operating characteristic (ROC) curve analyses and Kaplan–Meier analyses were performed to validate the signature in GEO cohorts and the triple-negative breast cancer (TNBC) subtype. We constructed an independent prognostic factor model with 11 TANRGs. The areas under the ROC curve (AUCs) of the TCGA training cohorts for 3-, 5-, and 7-year overall survival were 0.72, 0.73, and 0.73, respectively. The AUCs of the GEO test cohorts for 3-, 5-, and 7-year overall survival were 0.83, 0.89, and 0.94 (GSE25066) and 0.67, 0.69, and 0.73 (GSE58812), respectively. The proportion of immune subtypes differed among the different risk groups. The IC50 values differed significantly between risk groups and can be used as a guide for systemic therapy. The prognostic model developed by TANRGs has excellent predictive performance in BRCA patients. In addition, this feature is closely related to the prediction of survival, immune activity and treatment response in BRCA patients.

## Introduction

Breast cancer (BRCA) is a major global health problem because it threatens the healthcare of women worldwide and is the second leading killer of women's mortality from cancer^1^. In recent years, with the continuous development of treatments such as surgery, chemotherapy, radiotherapy, targeted therapy, and endocrine therapy, the survival rate of patients with invasive BRCA has improved^2^. Despite substantial progress, there are still some unresolved challenges, such as chemotherapy resistance, undetected distant metastases, treatment of triple-negative breast cancer (TNBC), and the lack of sufficient molecular targets. Moreover, due to individual heterogeneity, the overall prognosis of breast cancer remains suboptimal^3^. Therefore, developing accurate prognostic prediction strategies is necessary to improve clinical management. Compared to single indicators such as clinical and pathological parameters or individual gene expressions, comprehensive features that include multiple key characteristics appear to be more reliable tools for predicting prognosis.

Neutrophils are important inflammatory cells in the tumor immune microenvironment (TIME) and play a crucial role in tumorigenesis, progression, metastasis, immune regulation and response to therapeutics. Targeted modulation of tumor-associated neutrophil (TAN)-related pathways has the potential to be a promising therapeutic approach for BRCA^4,5^. Based on functional differences, TANs were divided into groups of N1, which inhibited tumors, and N2, which promoted tumors. N1 TANs include direct cytotoxic effects on tumor cells and inhibition of tumor metastasis. The N2 TANs can support tumor progression by promoting angiogenic switches, stimulating tumor cell motility, migration and invasion, and regulating other immune cells. Different TANs may coexist in tumors and adjust their metabolism to exert different or even opposite activities^6,7^. In melanoma, head and neck cancer and hepatocellular carcinoma patients, the density of TANs is of great importance for their prognosis^8^. The presence of TANs has been reported in several studies to be independently associated with low overall survival (OS), relapse-free survival, and disease-specific survival outcomes. Overall, TANs play a significant role in tumorigenesis and progression. However, research on the role of TAN-related genes in breast cancer is limited.

In this work, we constructed a multigenetic prognostic signature of differentially expressed genes (DEGs) associated with TAN. Consequently, we further found the relationship between TAN-related signature and the immune microenvironment. Our results indicate that this TAN-related model not only predicts the prognosis of breast cancer patients well, but also has clinical guidance significance for personalized immune treatment.

## Materials and methods

### Datasets and preprocessing

In our research, BRCA data were downloaded from TCGA (https://portal.gdc.cancer.gov/), including transcriptome RNA-sequencing data and clinical informativeness, comprising 104 normal and 1078 neoplastic tissues. The gene expression matrix dataset was downloaded from the Gene Expression Omnibus database (GEO, https://www.ncbi.nlm.nih.gov/geo/) and used for our validation. GSE25066 was generated by GPL96 (Affymetrix Human Genome U133A Array), including 508 tumor samples with BRCA. Both GSE58812 and GSE103091 were generated by GPL570 (Affymetrix Human Genome U133 Plus 2.0 Array), including 107 tumor samples with BRCA and 107 tumor samples with TNBC.

The patients from TCGA were defined as a training cohort, while the datasets from GSE25066, GSE58812 and GSE103091 were used for external validation. We used R (version 4.1.3) and the R Bioconductor package for data analysis.

### Preprocessing and profiling of tumor-associated neutrophil-related gene expression databases

A total of 2818 tumor-associated neutrophil-related genes (TANRGs) were retrieved from GeneCards (https://www.genecards.org/; accessed on 12 April 2023). We arranged the acquired genes according to their correlation scores and finally selected 466 genes with the relevance score > 4^9,10^.

A total of 466 TAN genes were analysed for comparison with the tumor tissues and the adjacent tissues to obtain differentially expressed TANRGs. We found 150 DEGs through the “limma” R package with the specific criteria of false discovery rate (FDR) < 0.05 and log2-fold change (FC) ≥ 1 in the TCGA cohort. A protein‒protein interaction (PPI) network of proteins encoded by DEGs was used for visualization by String (http://string-db.org) and Cytoscape. A *p* value of less than 0.05 was regarded as statistically significant.

### Establishment and validation of a TANRG prognostic signature

By univariate analysis, we identified TAN genes that were substantially related to prognosis. Then, we performed least absolute shrinkage and selector operator (LASSO) Cox regression (“glmnet” R package) to avoid overfitting^11^. We constructed prognostic models based on these prognostic TANRGs by developing multivariate Cox regression. The risk score formula was constructed to calculate the risk score: Risk score = ∑ni = ∑Coefi × xi, where xi denotes the normalized expression level of target gene i and Coefi denotes the regression coefficient. Based on the median risk score of the TCGA dataset, the 800 patients in the dataset were divided into a high-risk group and a low-risk group after excluding the samples with survival less than 30 days.

Kaplan‒Meier analysis and log-rank tests (using the "survival" R package) were applied to compare the OS of the two risk subgroups. The “survivalROC” R package was used to generate a time-dependent receiver operating characteristic (ROC) curve analysis to assess the predictive accuracy of the TANRG prognostic signature, and the areas under the curve (AUCs) at 3, 5, and 7 years were compared. In addition, progression-free survival (PFS) validation was performed for all TCGA- BRCA samples. We divided the patients into stage I-II and stage III-IV groups and performed OS validation with the same method. To analyse the differences in distribution between groups, principal component analysis (PCA) was implemented by the ‘prcomp’ function in the R. STATS package. A t-distribution stochastic neighbor embedding (t-SNE) analysis was performed by the R package Rtsne (https://github.com/jkrijthe/Rtsne).

### Univariate and multivariate Cox regression analysis

Assessment of the effectiveness of risk scores and clinical characteristics (age, stage, T-typing, N-typing, M-typing) on prognosis by univariate Cox regression. Multivariate Cox regression analysis was then conducted to determine which prognostic elements independently predicted patient survival. Adjusted p < 0.05 was regarded as statistically significant with the use of the ‘survival’ package. Afterwards, we explored a nomogram to forecast the probability of survival.

### Functional enrichment and pathway analysis

We divided BRCA patients into high- and low-risk subgroups based on median scores to further explore the biological processes associated with TANRGs. According to specific criteria (|log2FC|≥ 1 and FDR < 0.05), we extracted DEGs by comparing two risk groups. Based on these candidate DEGs. The "clusterProfiler" R package was applied to generate Gene Ontology (GO) enrichment analysis and Kyoto Encyclopedia of Genes and Genomes (KEGG) pathway analysis.

### Investigation of tumor-infiltrating immune cells and immune-related pathways

To further analyse the correlation between the risk and immune cell features, we calculated the immune infiltration status of the BRCA dataset from the TCGA project samples by currently accepted methods, including XCELL, QUANTISEQ, MCPcounter, EPIC, TIMER and CIBERSORT. The difference in the content of immune infiltrating cells between the two groups of the established model was analysed by using the Wilcoxon signed-rank test. Spearman correlation analysis was used to analyse the relationship between the infiltrated immune cells and risk scores. The resulting correlation coefficients are represented as a bar diagram. The threshold of significance was p < 0.05. The process was implemented with the R ggplot2 package.

Single-sample gene-set enrichment analysis (ssGSEA) was conducted using the "gsa" R package to calculate the scores of 16 tumor-infiltrating immune cells and 13 immune-related pathways^12^.

### Estimation of immune tumor microenvironment cell infiltration, immune checkpoints, and tumor mutation burden analysis

TME scores (ImmuneScore, StromalScore and ESTIMATEScore) were calculated for each BRCA patient using the "ESTIMATE" package^13–15^. In addition, the expression of immune checkpoints was used to examine the molecular correlation with prognostic characteristics. We assessed the correlation of this feature with tumor mutation burden (TMB) to compare the mutational load between the two groups.

### Estimation of drug sensitivity in the two risk groups

In the same way, we assessed the correlation between risk score and drug sensitivity with the R package "pRRophetic" and the CellMiner database. The R package “pRRophetic” was used to calculate the half-maximal inhibitory concentrations (IC50) of common chemotherapy agents. We used the Wilcoxon signed-rank test to compare IC50 values between the two risk groups. The CellMiner database (https://discover.nci.nih.gov/cellminer) was used to further forecast potential target agents (FDA-approved and in clinical trials) that might target the 20 TANRGs in the prognostic model^16,17^.

### Immunohistochemistry staining, methylation and survival probability

To evaluate the expression differences of prognostic model-related genes at the protein level, immunohistochemical images were used to detect CCL5, CCR7, EZR, IDH2, IL18, IL2RG, IL33, MAPK10 and MMP9 proteins in normal breast tissue and breast tumor tissue expression, which were downloaded from the HPA database (http://www.proteinatlas.org/) and analysed.

Moreover, we obtained boxplots for TANRG methylation levels and Kaplan‒Meier analysis survival probability between normal and tumor tissues in breasts through the UALCAN website (http://ualcan.path.uab.edu).

### Statistical analysis

Independent prognostic survival agents were ascertained by univariate and multifactorial Cox regression analyses. The predictive value of the model for OS was evaluated by Kaplan‒Meier analysis. The model’s predictive value for OS was assessed by performing Kaplan–Meier analysis. Time-dependent ROC curves were assessed for the predictive precision of the module. We performed all data analyses using R (version 4.1.0) and the R “Bioconductor” package. Unless otherwise indicated, p < 0.05 was considered statistically significant. P values are shown as follows: ns, not significant; *P < 0.05; **P < 0.01; ***P < 0.001.

### Ethics approval and consent to participate

The current study investigated publicly available data, and no ethical approval was needed.

## Results

### Identification of tumor-associated neutrophil-related DEGs in TCGA

**Figure 1:**
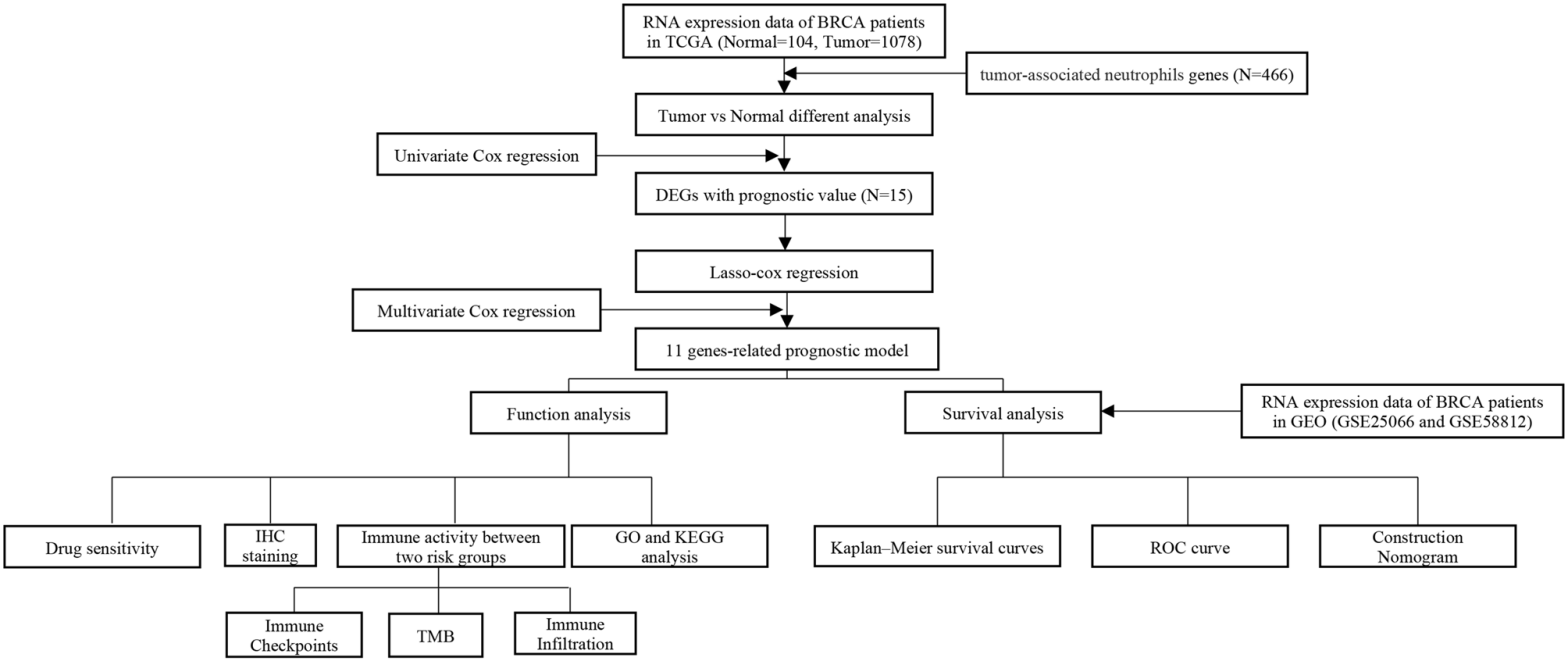
Flow diagram of the study.

**Figure 2:**
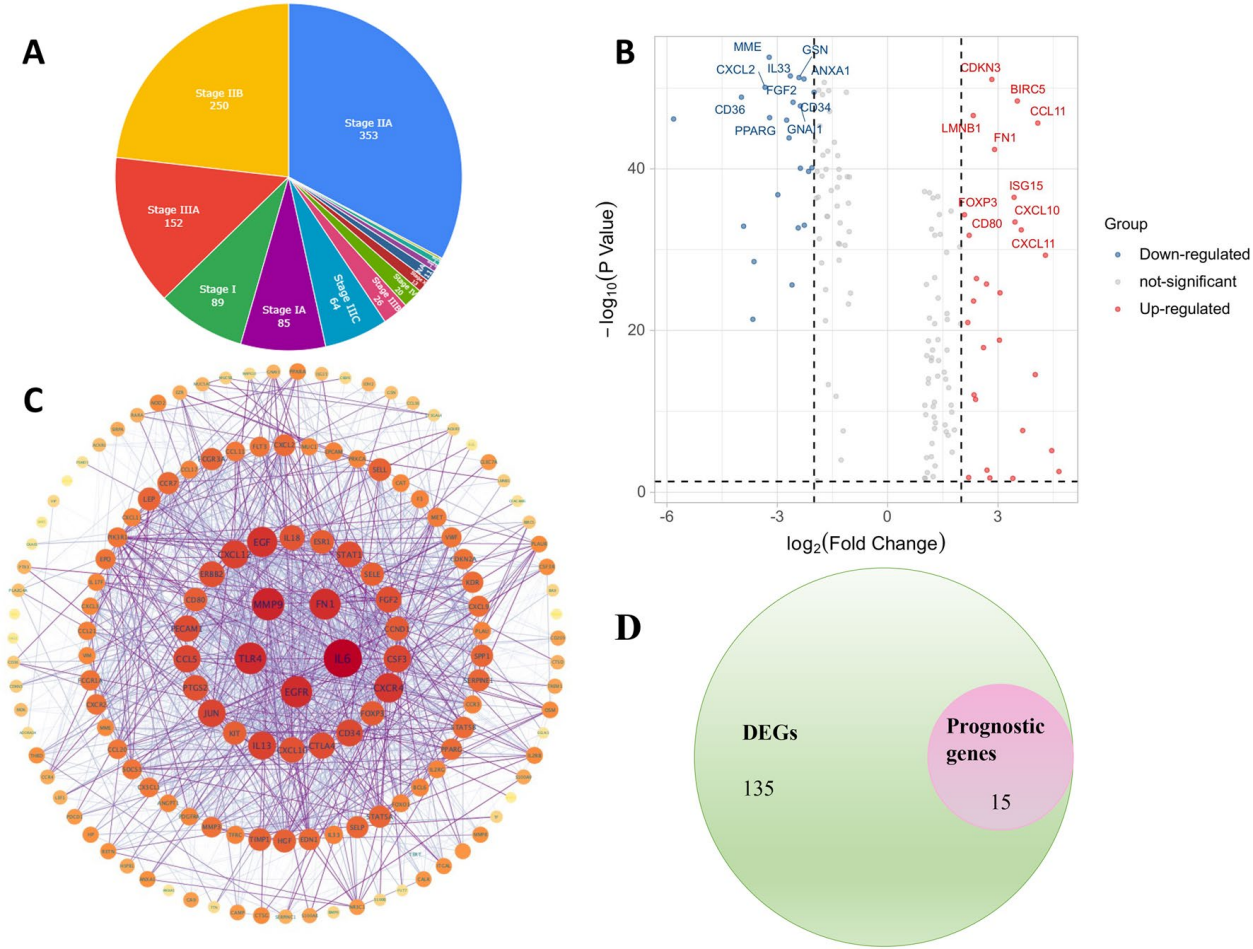
Characteristics of TCGA-BRCA patients and expression levels of the TANRGs. (**A**) Clinicopathological features of all patients included in this study. (**B**) Volcano plot of 150 differentially expressed genes. (**C**) PPI interaction analysis among the 150 DEGs with high confidence. (**D**) Venn diagrams of prognostic genes and DEG genes.

The flowchart of this study is shown in Fig. 1. We retrieved 1,182 breast RNA sequencing and clinical data from the TCGA database. The characteristics of those samples can be found in Fig. 2A. DEGs must meet the specific criteria of FDR < 0.05 and | log2FC |≥ 1. Finally, we identified 150 DEGs, 83 of which were upregulated and 67 of which were downregulated (Fig. 2B); detailed information can be found in Supplementary Table 1. In addition, we exhibit the interrelationships of those 150 DEGs via the PPI network and Cytoscape application in Fig. 2C.

### Construction of a TANRG-related prognostic model

**Figure 3:**
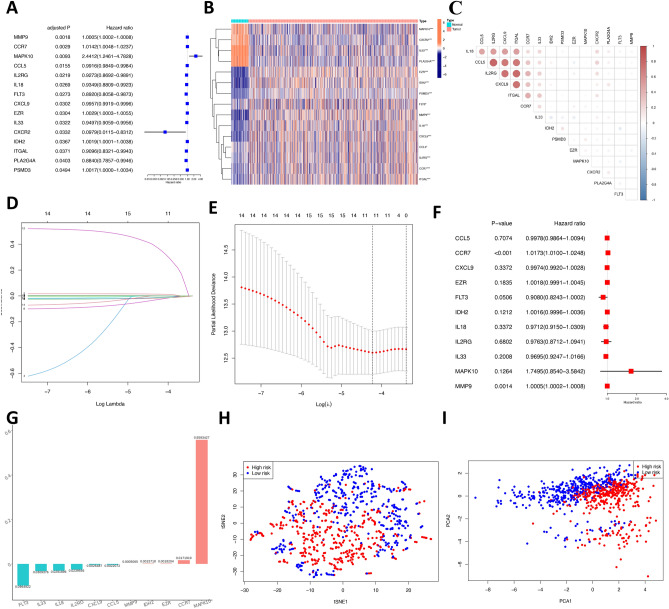
Construction of the TANRG prognostic signature. (**A**) Univariate Cox regression analysis of 15 TANRGs and all genes with *p* < 0.05. (**B**) Heatmap of prognostic gene expression between normal and tumor breast tissue. (**C**) The corrplot results of 15 prognostic genes. (**D**,**E**) LASSO regression analysis. (**F**) Multivariate Cox regression analysis of 11 TANRGs and two genes with *p* < 0.05. (**G**) Gene coefficient histogram of 11 TANRGs. (**H**) Two-dimensional projection by t-SNE analysis in the training cohort. (**I**) Score plot for the principal component analysis (PCA) in the training cohort.

First, we identified that 15 genes were related to prognosis by conducting univariate Cox regression under the criteria of p < 0.05, and Venn diagrams of prognostic genes and DEG genes are shown in Fig. 2D. Nine genes (CXCL2, PLA2G4A, FLT3, ITGAL, IL2RG, IL18, IL33, CCL5 and CXCL9) had a protective effect (HRs < 1), and the other six genes (MMP9, PSMD3, IDH2, EZR, CCR7 and MAPK10) may be risk factors with HRs > 1 (Fig. 3A).

The gene expression heatmap of 15 prognostic genes in normal breast tissue and BRCA tissue (Fig. 3B) showed that the MAPK10, CXCL2, IL33 and PLA2G4A genes were highly expressed in normal tissue, while the remaining 11 genes were expressed at low levels in normal tissue. The corrplot results of 15 prognostic genes are shown in Fig. 3C.

Then, we eliminated the overlapping problem of those 15 candidate genes through the LASSO test (Fig. 3D,E). Afterward, the prognostic signature was constructed by those 11 candidate genes by performing a multivariate Cox regression analysis. Detailed information can be found in Fig. 3F, and there were two genes (CCR7 and MMP9) with *p* < 0.05. The gene coefficients of 11 modelling candidate genes can be found in Fig. 3G and Table 1.

**Table 1:** The genes involved in the signature and their coefficients.

Gene	Coefficient
FLT3	− 0.0964922
IL33	− 0.0309376
IL18	− 0.0291896
IL2RG	− 0.0239556
CXCL9	− 0.0026487
CCL5	− 0.0022072
MMP9	0.0005065
IDH2	0.0015718
EZR	0.0018204
CCR7	0.0171919
MAPK10	0.5593427

TANRG risk score = (-0.0964922 * expression of FLT3) + (-0.0309376 * expression of IL33) + (− 0.0291896 * expression of IL18) + (-0.0239556 * expression of IL2RG) + (-0.0026487 * expression of CXCL9) + (-0.0022072 * expression of CCL5) + (0.0005065 * expression of MMP9) + (0.0015718 * expression of IDH2) + (0.0018204 * expression of EZR) + (0.0171919 * expression of CCR7 + (0.5593427 * expression of MAPK10). The BRCA could be separated into the high- and low- groups based on the median value (1.103) of risk score in the training set. We can see from t-SNE mappings and PCA (Fig. 3H and I) that patients with two risk scores formed two different clusters based on the distribution of the risk score, survival time, survival status, and relevant expression standards of these genes.

**Figure 4:**
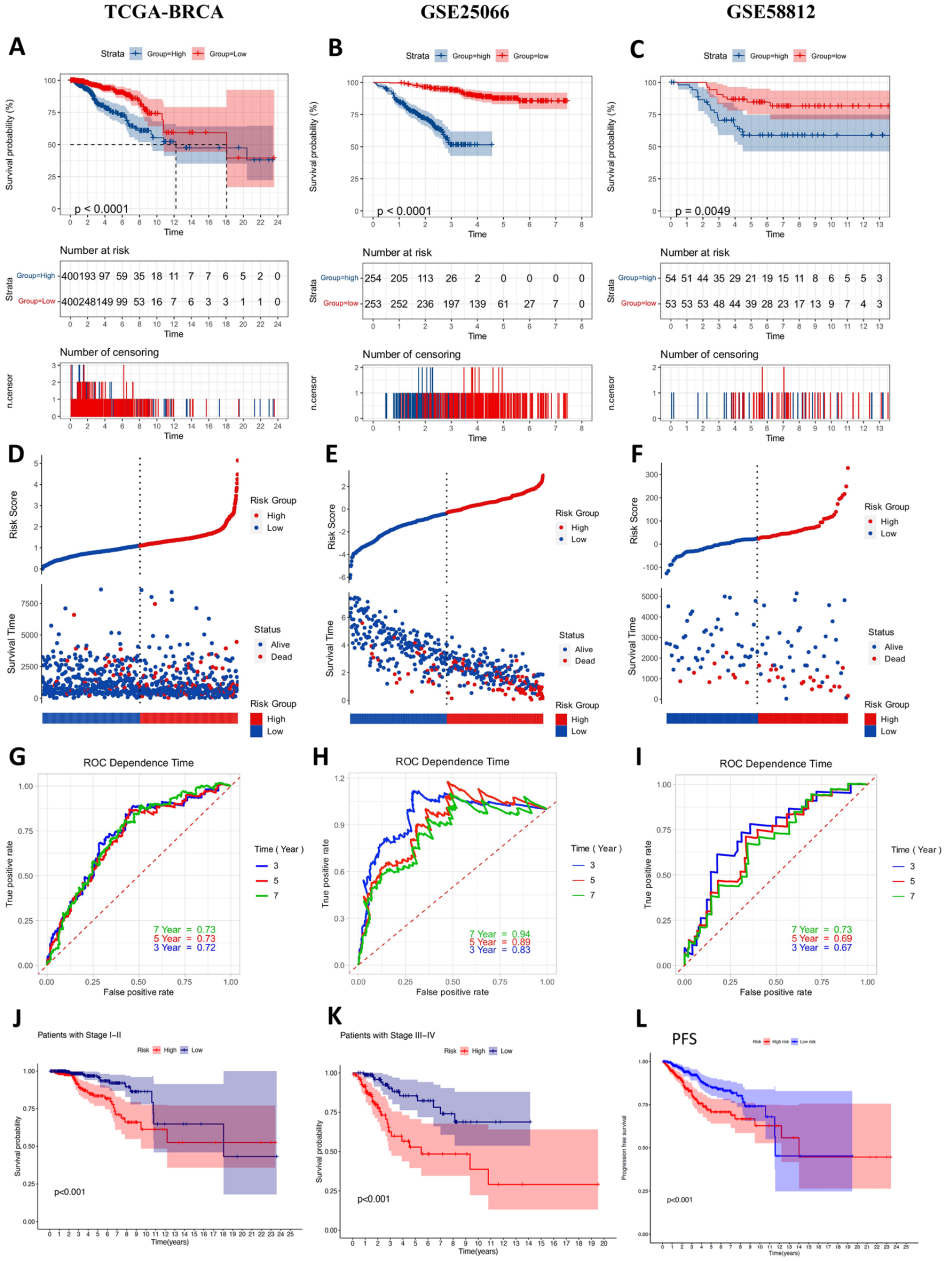
The prognostic performance of the 11-gene signature in the training cohort and validation cohort. (**A**) Kaplan‒Meier curves for the overall survival of patients in the high- and low-risk groups in the TCGA cohort, (**B**) GSE25066 cohort, (**C**) and GSE58812 cohort. (**D**) The distribution of the risk scores and scatter plots showed whether the samples were alive in the TCGA cohort, (**E**) GSE25066 cohort, (**F**) and GSE58812 cohort. (**G**) ROC curves in TCGA, (**H**) GSE25066 cohort, (**I**) and GSE58812 cohort, respectively. (**J**) Overall survival validation in stage I-II TCGA-BRCA patients (**K**) and stage I-II TCGA-BRCA patients. (**L**) Progression-free survival validation in TCGA-BRCA patients.

The patients in the low-risk subgroup had longer OS times than those patients in the high-risk subgroup according to the Kaplan‒Meier plot in the TCGA cohort (*p* < 0.0001; Fig. 4A). Figure 4D shows the risk score distribution and survival status in two risk subgroups of patients in the TCGA database. The patient’s survival time decreased, and more patients died as the risk score increased. Subsequently, the predictive accuracy of this TANRG-related prognostic signature was assessed by generating time-dependent ROC curve analyses. The AUCs of the TCGA cohorts at 3, 5, and 7 years were 0.72, 0.73, and 0.73, respectively (Fig. 4G), which indicated that the TAN-related signature had moderate predictive power.

In addition, TCGA-BRCA was divided into two groups, stage I-II and III-IV, for OS validation, and the results showed that OS was longer in the low-risk group than in the high-risk group in both the stage I-II group (*p* < 0.001; Fig. 4J) and the stage III-IV group (*p* < 0.001; Fig. 4K). Moreover, PFS was also superior in the low-risk group than in the high-risk group of TCGA-BRCA patients (*p* < 0.001; Fig. 4L).

### Validation of the TANRG-related prognostic model in the GEO cohort and subtypes cohort

To evaluate the ability and accuracy of the TANRG-related signature, we validated it using the same risk formula in the validation cohort from the GEO dataset (GSE25066 and GSE51822). The validation set of GSE25066 was segregated into high (*N* = 254) and low (*N* = 253) risk groups. K-M analysis showed that patients in the high-risk group also had a worse prognosis than those in the low-risk group (*p* < 0.0001, Fig. 4B). Each patient’s risk status and survival outcome are shown in Fig. 4E. The AUCs of the prognostic signature at 3, 5, and 7 years were 0.83, 0.89, and 0.94, respectively (Fig. 4H). The other validation set of GSE58812 was segregated into high (*N* = 54) and low (*N* = 53) risk groups. Consistent with our previous analysis, K-M analysis showed that patients in the high-risk group also had a worse prognosis than those in the low-risk group (*p* = 0.0049, Fig. 4C). Each patient’s risk status and survival outcome are shown in Fig. 4F. The AUCs of the prognostic signature at 3, 5, and 7 years were 0.67, 0.69, and 0.73, respectively (Fig. 4I).

**Figure 5:**
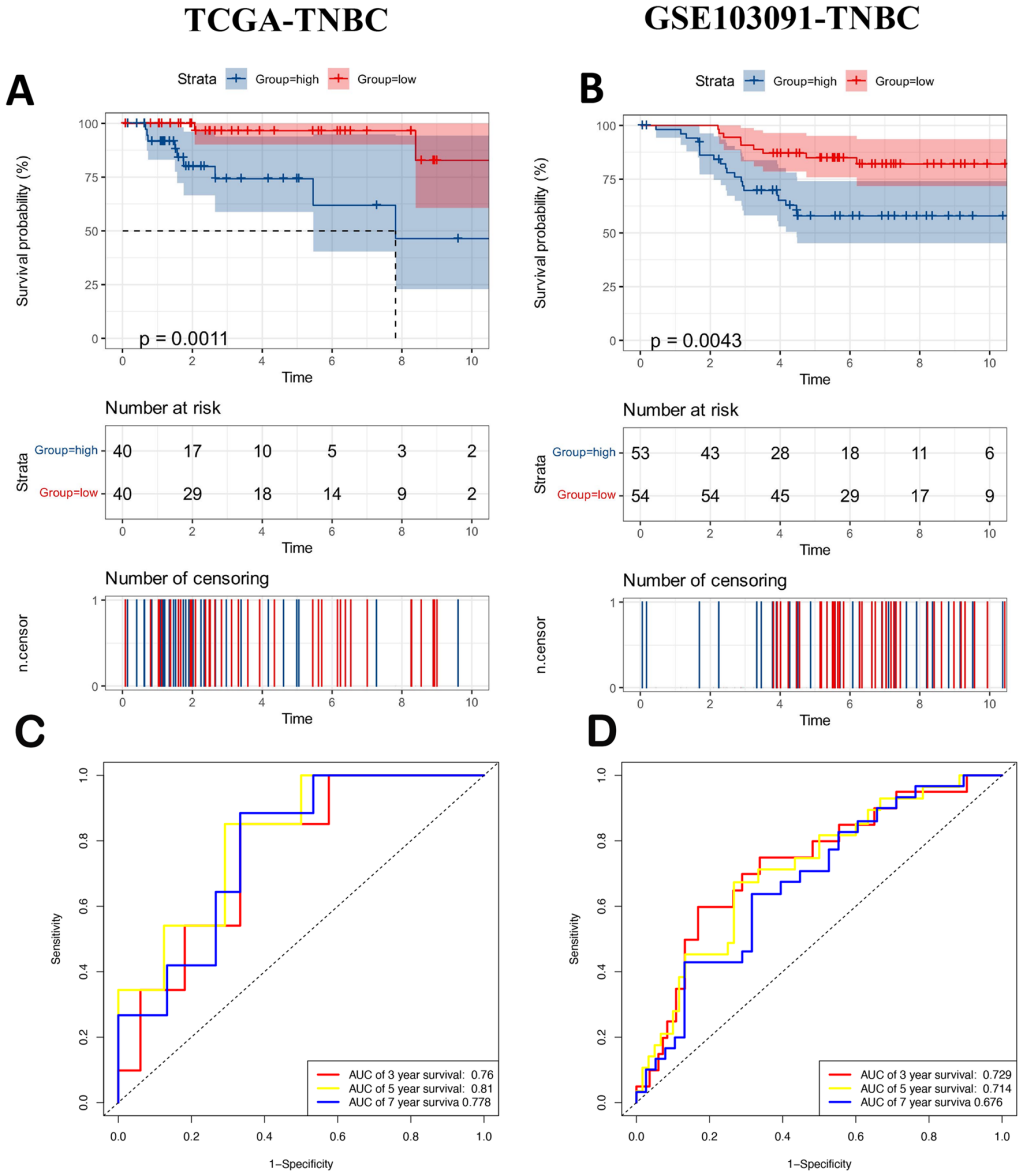
The prognostic performance of the 11 TANRG signature in the TCGA-TNBC and GEO-TNBC test cohorts. (**A**) Kaplan‒Meier curves for the overall survival of patients in the high- and low-risk groups in the TCGA-TNBC cohort and (**B**) the GSE103091-TNBC cohort. (**C**) ROC curves in the TCGA-TNBC and (**D**) GSE103091-TNBC cohorts.

Breast cancer is widely accepted as a heterogeneous disease, and current treatment approaches consider its various subtypes. A signature designed for the entire spectrum of breast cancer may have limited utility. Therefore, we validated the ability and accuracy of the signature using the same risk formula in the subtypes cohort including triple-negative breast cancer (TNBC), Luminal and Her-2 from TCGA cohort. In addition, we have also found GEO data for TNBC. TCGA-TNBC was segregated into high (*N* = 40) and low (*N* = 40) risk groups. K-M analysis showed that patients in the low-risk group also had a better prognosis than those in the high-risk group (*p* = 0.0011, Fig. 5A). The AUCs of the prognostic signature at 3, 5, and 7 years were 0.76, 0.81, and 0.78, respectively (Fig. 5C). GSE103091-TNBC was segregated into high (*N* = 53) and low (*N* = 54) risk groups. K-M analysis showed that patients in the low-risk group also had a better prognosis than those in the high-risk group (*p* = 0.0043, Fig. 5B). The AUCs of the prognostic signature at 3, 5, and 7 years were 0.73, 0.71, and 0.68, respectively (Fig. 5D). However, the K-M curve did not differentiate the survival differences between the high and low-risk groups within the HER-2 and Luminal subtypes (Figs. S1, 2), suggesting that the model may be more suitable for the TNBC.

### Univariate and multivariate Cox regression of clinical characteristics

**Figure 6:**
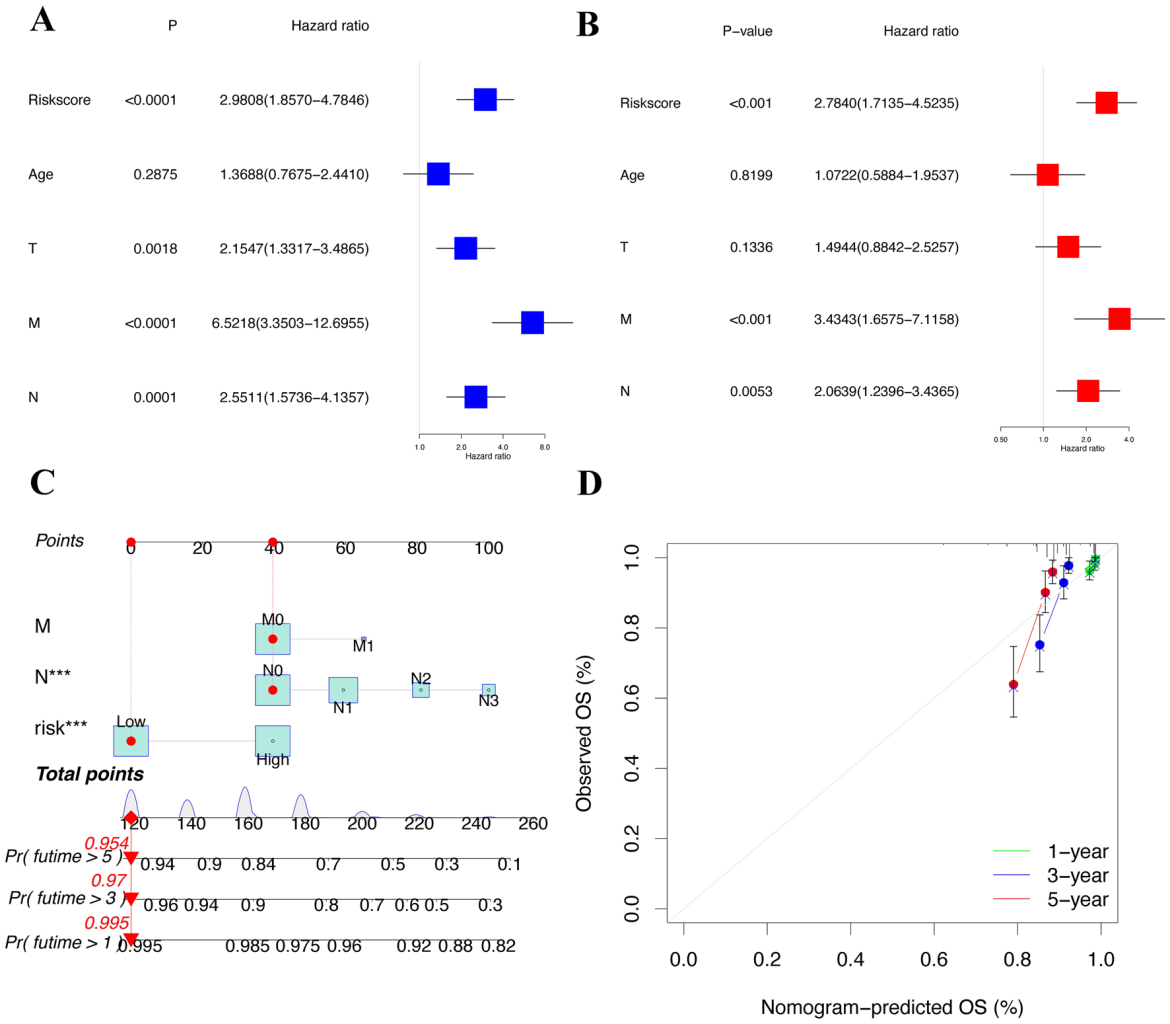
Independent prognostic predictors of the risk assessment model in the TCGA cohort. (**A**) Forest plot of univariate analysis. (**B**) Forest plot of multivariate analysis. (**C**) Nomogram to predict the 3-, 5-, and 7-year overall survival rates. (**D**) Calibration curves of the nomogram for 3-, 5-, and 7-year overall survival prediction.

We demonstrated that the risk score (*p* < 0.001, HR = 2.98, 95% CI [1.86–4.78]), clinical stage (*p* < 0.001, HR = 3.13, 95% CI [2.02–4.85]), pT stage (p = 0.0018, HR = 2.15, 95% CI [1.33–3.49]), pN stage (p = 0.0001, HR = 2.55, 95% CI [1.57–5.14]), and pM stage (*p* < 0.0001, HR = 6.52, 95% CI [3.35–12.70]) showed significant differences by univariate Cox regression analysis (Fig. 6A), whereas the risk score (*p* < 0.001, HR = 2.87, 95% CI [1.77–4.67]), clinical stage (*p* = 0.0245, HR = 2.18, 95% CI [1.11–4.28]) and pM stage (*p* = 0.0111, HR = 2.63, 95% CI [1.25–5.54] still significant differences by multivariate Cox regression analysis (Fig. 6B) The detailed results of univariate and multivariate Cox regression analyses can be found in Table 2.

**Table 2:** Univariable and multivariable analyses for each clinical feature.

Clinical feature	Univariate analysis				Multivariate analysis			
	HR	HR_95L	HR_95U	*P*	HR	HR_95L	HR_95U	*P*
Riskscore (high-risk/low-risk)	2.981	1.857	4.785	< 0.0001	2.873	1.765	4.674	< 0.0001
Age (≤ 45/ > 45)	1.369	0.768	2.441	0.2875	1.103	0.604	2.012	0.7500
Stage (I–II/III–IV)	3.134	2.025	4.852	< 0.0001	2.175	1.105	4.280	0.0245
T (I–II/III–IV)	2.155	1.332	3.486	0.0018	0.982	0.528	1.824	0.9532
M (0/1)	6.522	3.350	12.696	< 0.0001	2.628	1.247	5.541	0.0111
N (0/1–3)	2.551	1.574	4.136	0.0001	1.558	0.864	2.810	0.1402

*T* tumor, *N* lymph node, *M* metastasis, *HR* hazard ratio.

### Construction of the nomogram

We constructed a BRCA prognostic nomogram together with independent prognostic factors, including stage, pM and risk score. The prognostic nomogram predicted patient survival at 3, 5, and 7 years (Fig. 6C), and calibration curves demonstrated the reliability of the nomogram in forecasting survival prognosis (Fig. 6D).

### Functional enrichment analyses

To further explore the functional differences between the two risk subgroups, we obtained 518 DEGs from the two risk groups by using the “limma” R package under the specific criteria (|log2FC|≥ 1 and FDR < 0.05). GO and KEGG functional enrichment analyses were generated to investigate the potential functions between the two risk subgroups in the TCGA database.

**Figure 7:**
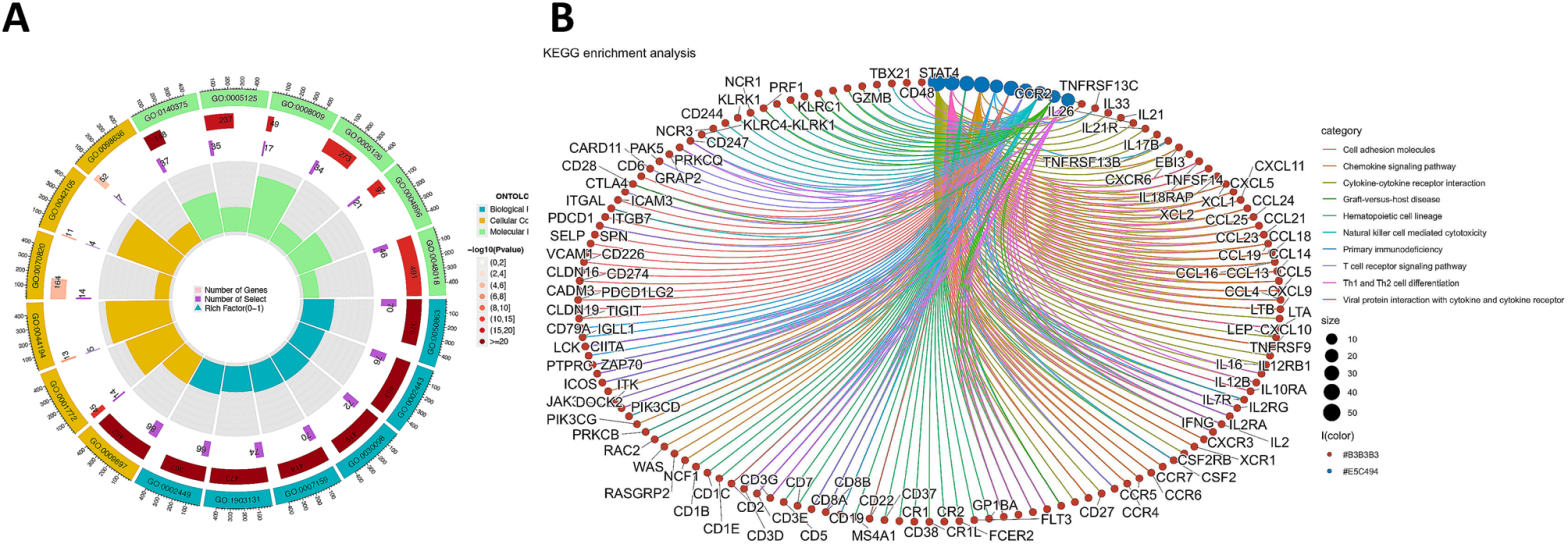
Gene ontology (GO) and Kyoto encyclopedia of genes and genomes (KEGG) enrichment analyses of the DEGs between the two risk groups in TCGA cohort. (**A**) GO enrichment analysis of the 518 DEGs. (**B**) KEGG enrichment analysis of the 518 DEGs.

The GO analysis showed that the DEGs were equally concentrated in regulation of T-cell activation, leukocyte-mediated immunity, lymphocyte differentiation, leukocyte cell‒cell adhesion, mononuclear cell differentiation, lymphocyte-mediated immunity, external side of plasma membrane, etc. (Fig. 7A). KEGG functional enrichment analysis showed that the DEGs were related to cytokine‒cytokine receptor interaction, viral protein interaction with cytokine and cytokine receptor, hematopoietic cell lineage, chemokine signaling pathway, primary immunodeficiency, cell adhesion molecules, T-cell receptor signaling pathway, etc. (Fig. 7B).

### Investigation of immunity factors in risk groups

**Figure 8:**
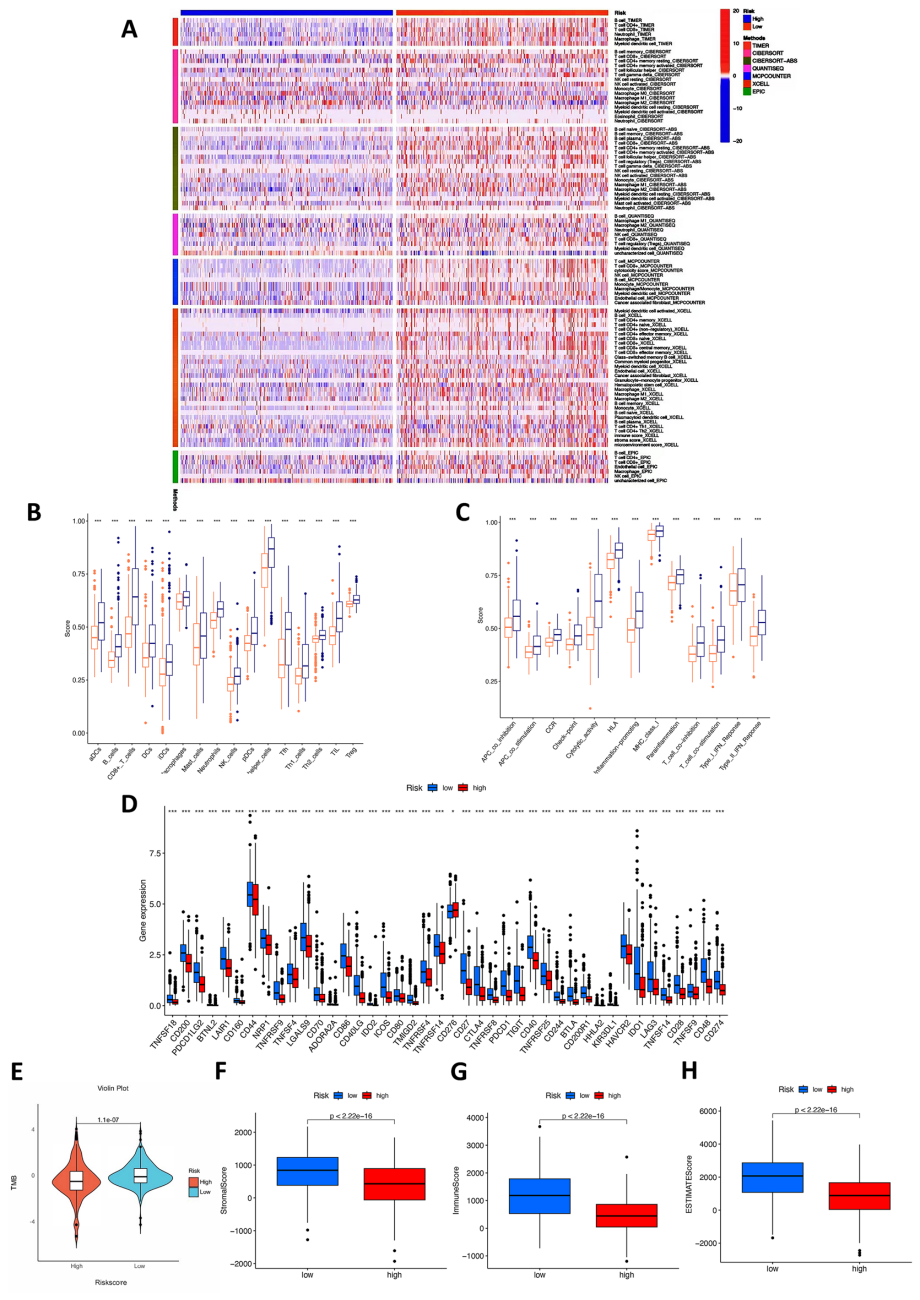
Investigation of tumor immune factors. (**A**) The immune cell heatmap of risk groups in different platforms. (**B**) Comparison of the enrichment scores of 16 types of immune cells between the low- (blue box) and high-risk (red box) groups in the TCGA cohort of ssGSEA. (**C**) Comparison of the enrichment scores of 13 immune-related biological processes between the low- (blue box) and high-risk (red box) groups in the TCGA cohort of ssGSEA. (**D**) Immune checkpoints, (**E**) tumor mutation burden, (**F**) stromal scores, (**G**) immune scores, and (**H**) ESTIMATE scores in the high- and low-risk groups.

As shown in the immune cell heatmap in Fig. 8A, the infiltration degree of immune cells was stronger in the low-risk score group than in the high-risk score group on various platforms (*p* < 0.05). The detailed results are listed in Supplementary Table 2.

To further investigate the relationship between BRCA prognosis and immune status, we assessed the immune-related function and immune cell infiltration score using ssGSEA. The results also showed that all 16 types of immune cells (iDCs, B cells, DCs, aDCs, CD8 + T cells, T helper cells, pDCs, NK cells, mast cells, neutrophils, pDCs, TIh cells, TILs, Th1 cells, Th2 cells, and Tregs) were significantly correlated with the TAN-related prognostic signature (*p* < 0.001), and the low-risk group had higher levels of immune cell infiltration (Fig. 8B).

Meanwhile, all 16 types of immune-related pathways were significantly different between the low- and high-risk groups in the TCGA database (*p* < 0.001). All of these results showed that the low-risk group had a higher immune infiltration status (Fig. 8C).

### TMB, immune checkpoint analysis and TME cell infiltration between the two subgroups

We found a relationship between the TAN-related signature and the immune response in the above results. Overexpression of immune checkpoints is often associated with immune escape mechanisms, and immunotherapy targeting immune checkpoints has become a new direction of current research. In addition, immune checkpoint inhibitors (ICIs) are administered for treating BRAC in clinical practice. We investigated whether the risk model was related to ICI-related biomarkers and discovered that the expression of immune checkpoints, including PD-1 (PDCD1), PD-L1 (CD274), and CTLA-4, as well as some novel immune checkpoints, such as CD80, CD28 and CD48, was significantly upregulated in the low-risk group (p < 0.001; Fig. 8D). In addition, only CD276 was downregulated in the low-risk group (p < 0.05), suggesting a potential role of the signature model in predicting immune responses to immunotherapy in BRCA patients^18^.

The efficacy of tumor immunotherapy can be monitored via the TMB score. Therefore, the relationship between TMB and the risk signature was determined. The results showed that the risk scores were negatively associated with the expression level of TMB (p < 0.001; Fig. 8E). The correlation between the risk score and the TME score was investigated, and a significant negative association between the immune score, stromal score, and ESTIMATE score and risk score was found (Fig. 8F–H).

The results showed that the high- and low-risk groups responded differently to immunotherapy based on the TANRG-related prognostic model, and the low-risk group tended to be more sensitive to immunotherapy than the high-risk group.

### TANRG-related prognostic model predicts drug sensitivity

To further enhance the clinical value of the TANRG-related prognostic model for treating BRCA, we estimated the efficacy of chemotherapy and potential agents for BRCA patients with the “pRRophetic” and CellMiner databases. We assessed the IC50 for common chemotherapeutic agents against BRCA with the “pRRophetic” algorithm and compared the IC50 between the high- and low-risk groups. The IC50 value was the opposite of the sensitivity of the drugs.

**Figure 9:**
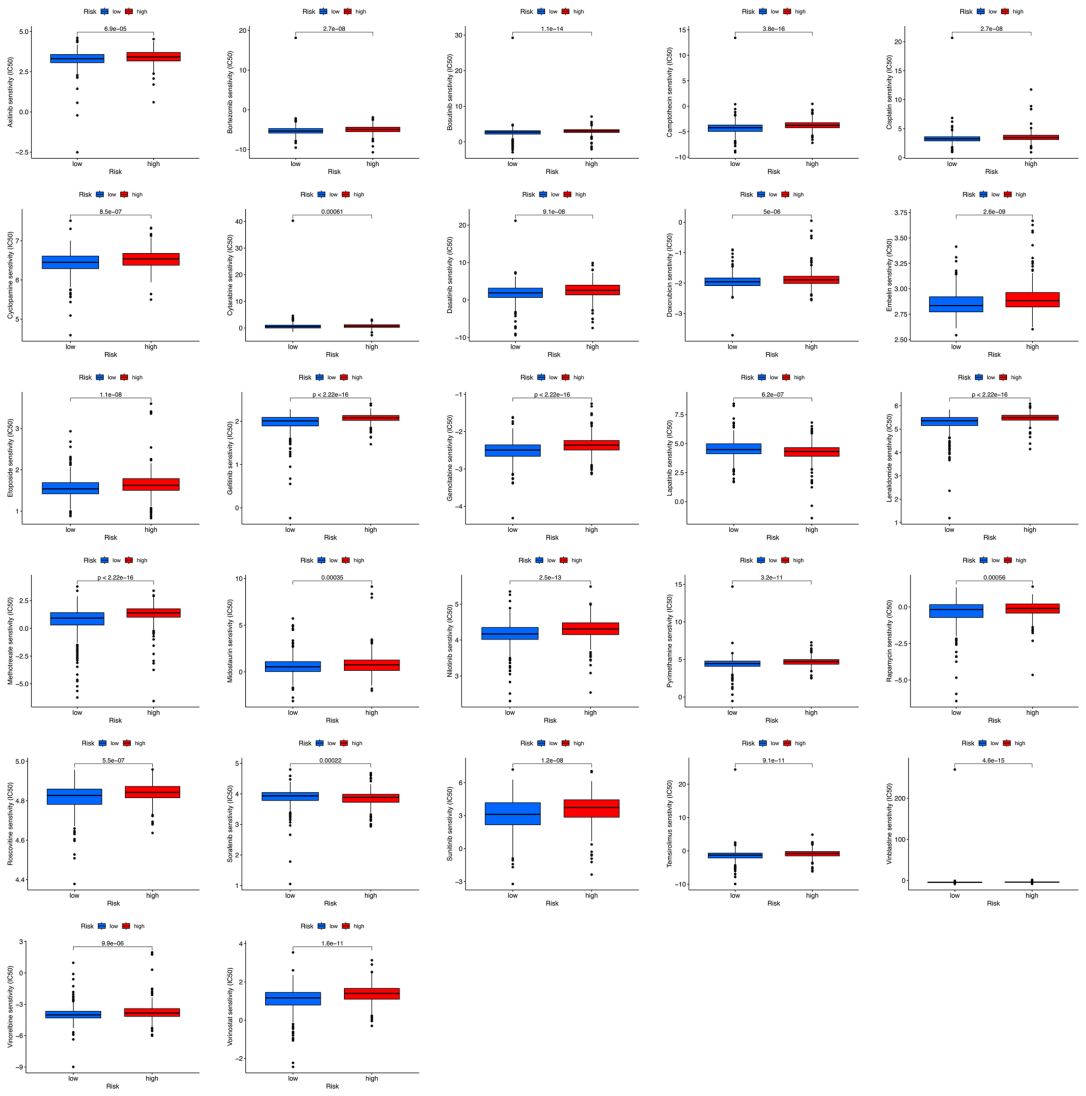
Comparison of the IC50 values of drugs in the two risk groups.

We showed that a low risk score was associated with a lower IC50 of chemotherapeutics such as axitinib, bortezomib, bosutinib, camptothecin, cisolatin, cyclopamine, cytarabine, dasatinib, doxorubicin, embelin, etoposide, gefitinib, gemcitabine, lenalidomide, methotrexate, midostaurin, nilotinib, pyrimethamine, banamvcin, roscovitine, sunitinib, temsirolimus, vinblastine, vinorelbine, and vorinostat (p < 0.001) compared with the high risk score group. These results demonstrated that patients in the low-risk group were relatively sensitive to these agents. In contrast, the high-risk group was associated with lower IC50 levels for lapatinib and sorafenib than the low-risk group (Fig. 9). This suggests that patients in the high-risk group are relatively more sensitive to the two targeted agents.

**Figure 10:**
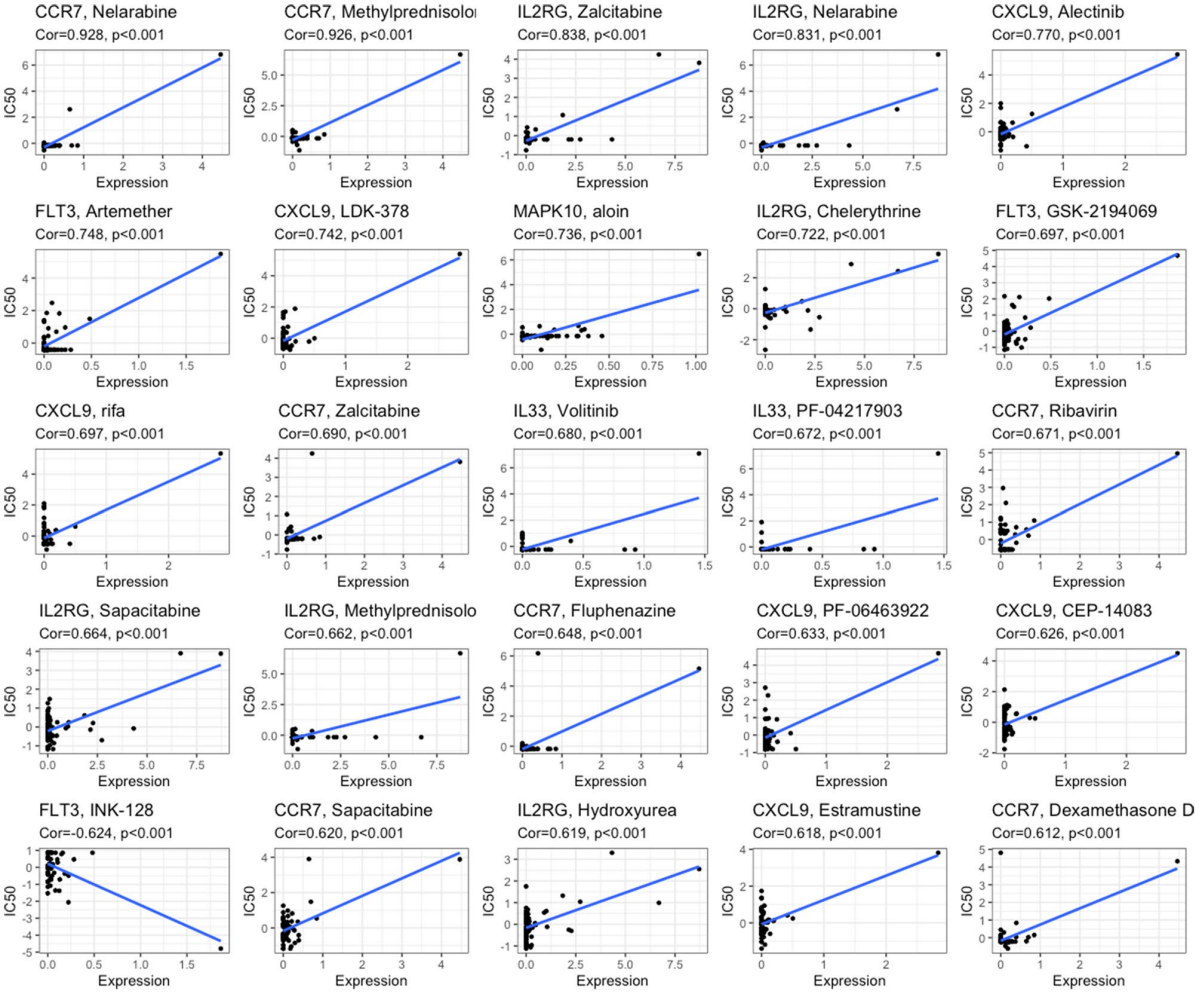
Sensitivity correlation analyses of the TANRGs and potential drugs according to the CellMiner database.

Moreover, 136 drugs targeting TANRGs were significantly available for treating BRCA according to CellMiner (p < 0.001, Supplementary Table 3). We selected the top 25 by *p* value to plot. As shown in Fig. 10, high expression of CCR7, IL2RG, CXCL9 and MAPK10 was associated with elevated drug sensitivity of cancer cells, while high expression of FLT3 was associated with diminished drug sensitivity of cancer cells to one drug (INK-128). Based on these findings, the risk score can guide patients in receiving more appropriate drug treatment.

### Immunohistochemistry staining, methylation and survival probability

**Figure 11:**
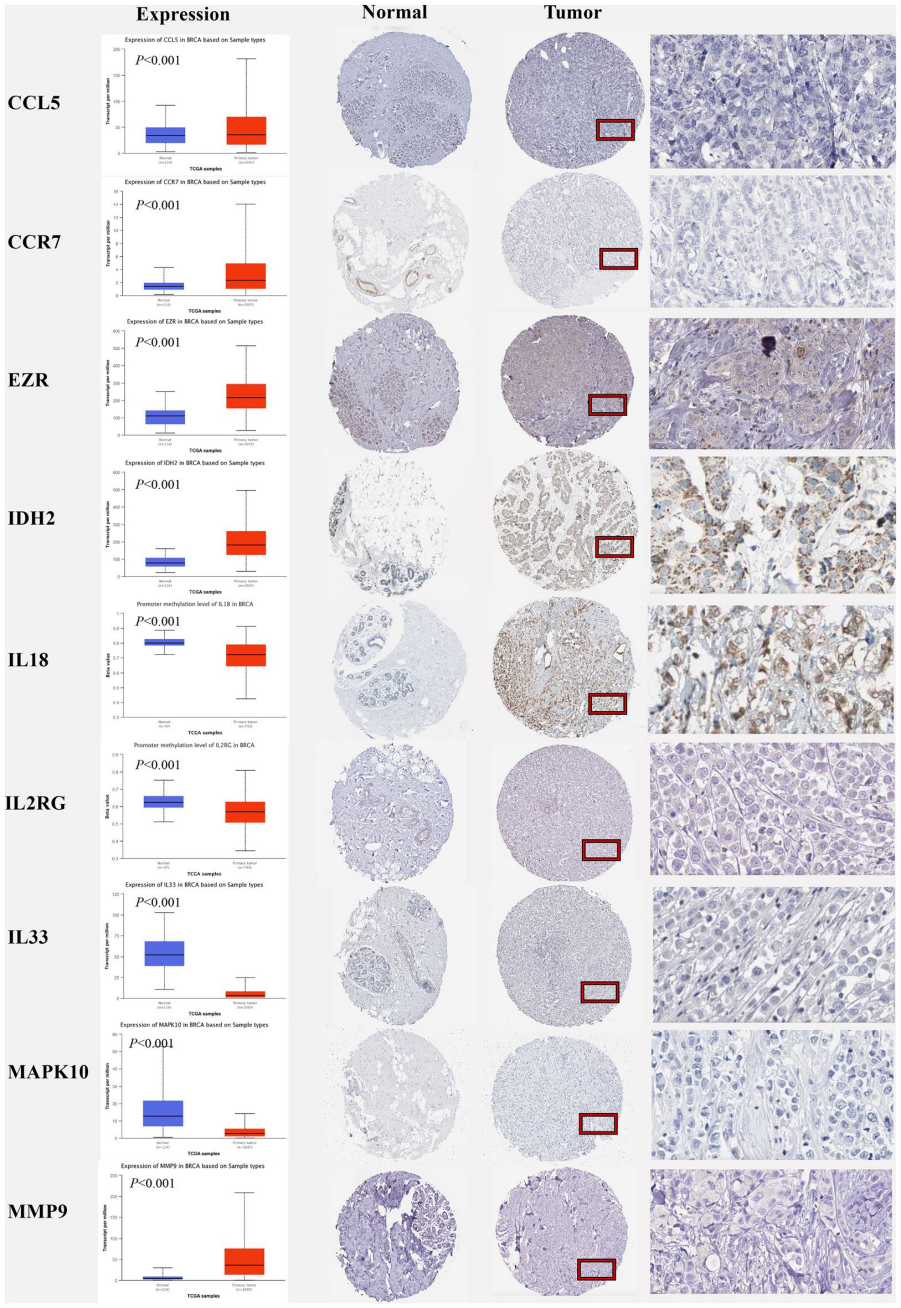
Comparison of gene expression and immunohistochemistry images between normal and tumor tissues.

To assess the expression of TANRGs of the prognostic module at the protein level, we analysed the immunohistochemical results provided by the HPA database and compared the results with the corresponding gene expression data provided by TCGA. As shown in Fig. 11, the results of data analysis from both databases were consistent. CCR7, EZR, IDH2 and MMP9 were highly expressed in breast tumor tissues, whereas they were expressed at low levels in normal breast tissues (*p* < 0.001). In contrast, IL18, IL2RG, IL33 and MAPK10 were highly expressed in normal breast tissues, whereas they were expressed at low levels in breast tumor tissues (*p* < 0.001).

**Figure 12:**
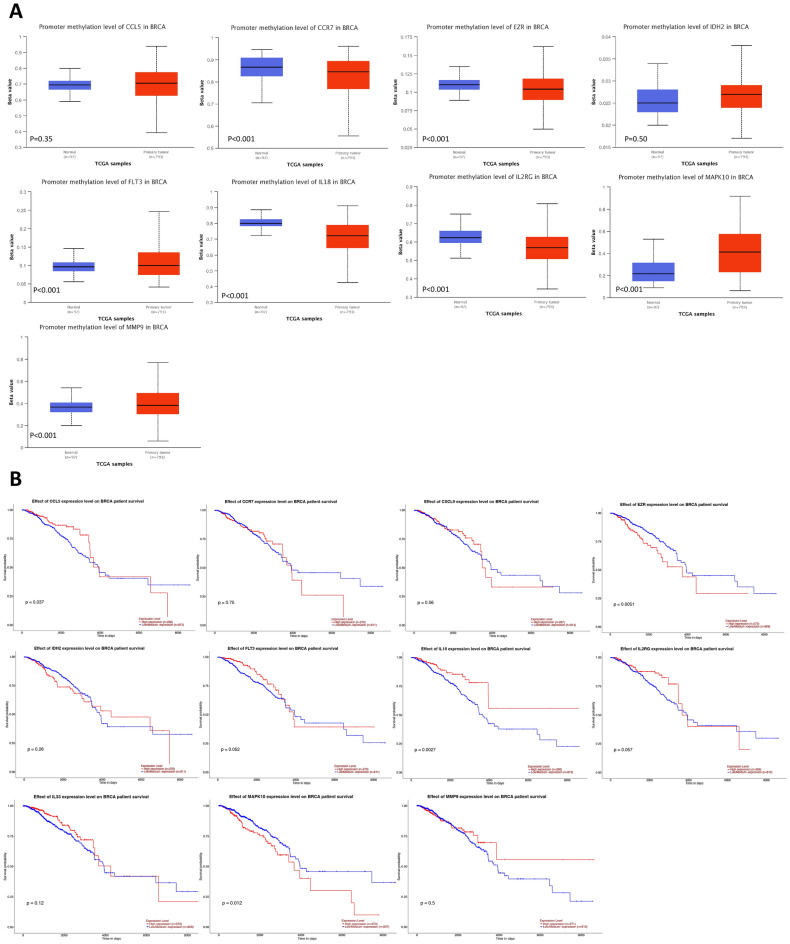
Methylation and survival probability. (**A**) Comparison of methylation between normal and tumor tissues. (**B**) Survival probability between low and high gene expression in BRCA.

In addition, we evaluated methylation in normal and tumor tissues of the breast of TCGA data from UALCAN. The results showed that CCL5 and IDH2 were not significantly differentially methylated in BRCA, while CCR7, EZR, IL8, and IL2RG were hypermethylated in normal tissues, and FLT3, MAPK10, and MMP9 were hypermethylated in breast tumor tissues (Fig. 12A).

All the 11 gene in the TANRG prognostic model was divided into high and low expression groups according to gene expression, and its survival probability in BRCA is shown in Fig. 12B, only four genes, including CCL5, EZR, IL18 and MAPK10, were significant in OS (*p* < 0.05). Among them, CCL5, IL18 had worse OS in the low expression group, while EZR and MAPK10 had better OS in the low expression group.

## Discussion

BRAC is one of the most common malignancies threatening women's health worldwide. Depending on the clinical and molecular characteristics of the breast tumor, patients receive different treatment options. In the rapidly evolving field of personalized medicine research, predictive biomarkers are an essential tool to select patients most likely to benefit from various treatments and to be able to provide the right treatment to the right patient, avoiding overdosing or unnecessary treatment. The importance and function of neutrophils in different types of carcinomas has changed dramatically in the last decade, as neutrophils used to be considered mere bystanders in the TIME. Studies have shown that neutrophils accumulate in large numbers in the peripheral blood of people with cancer, especially in patients with late-stage disease, and that higher ratios of circulating neutrophils to lymphocytes have become reliable biomarkers of poor prognosis in patients^8,19^. Therefore, we constructed a model based on TAN-related genes to assess the different prognoses and immune infiltration statuses of patients and drug sensitivity, enabling BRAC patients to receive more precise treatment.

The prognostic model in this study incorporated 11 TAN-associated genes (CCL5, CCR7, CXCL9, EZR, FLT3, IDH2, IL18, IL2RG, IL33, MAPK10 and MMP9). A number of studies have highlighted the critical role of these TANRG genes in the development, progression or metastasis of breast tumors. CCL5 was positively associated with axillary lymph node metastasis and poor prognostic predictors of BRCA, which was mainly mediated through CCR5/Treg cells^20^. CCR7 has a crucial effect on the development of BRCA. CCR7 and its receptors have been specifically reported to be stimulatory to breast carcinogenesis and to be responsible for a novel action in triggering lymphangiogenesis. In addition, CCR7 is also involved in tumor cell invasion, metastasis, development and epithelial mesenchymal transition, as well as in lymph node metastasis, invasion and migration^21^. CXCL9 is highly expressed in triple-negative BRCA tissues, and it can alter the TIME by stimulating MHC-II activity through JAK/STAT signaling^22^. High EZR gene expression has been reported to be connected with poorer OS in BRCA patients^23^. High expression of either FLT3 or IDH2 is an independent adverse prognostic factor in BRCA^24,25^, and patients with high FLT3 expression have a greater responsiveness to combination treatments^24^. Increased IL18 expression levels in nasopharyngeal carcinoma lead to poor prognosis^26^. IL-33 promotes endocrine resistance in BRCA by inducing stem cell properties^27^, and in addition, IL33 of fibroblast-derived origin promotes breast tumor metastasis to the lung by altering the TIME^28^. A few miRNAs can promote BRCA cell proliferation, metastasis and infiltration and inhibit BRCA cell apoptosis by inhibiting MAPK10^29^. MMP9 overexpression is also an important predictor of prognosis in BC patients. MMP9 overexpression is significantly associated with higher clinical stage, larger tumor size, and poorer prognosis in BC patients^30^. All of these genes are expected to be potential clinical biomarkers for the prognosis of BRCA patients and for predicting the efficacy of immunotherapy.

In this study, we constructed a prognostic model of 11 TANRG genes as a prognostic biomarker and evaluated it to predict prognosis, immune infiltration status, and sensitivity to drug therapy in two different risk groups. The results showed that patients in the low-risk group survived longer than those in the high-risk group, including OS and PFS. The reliability of our prognostic model and findings was confirmed by using ROC curves in TCGA, PCA and t-SNE analysis and ROC curves from three independent GEO datasets. Additionally, tumor stage, risk score and pM were all good independent survival indicators after adjusting for clinical parameters. The above confirmed the good predictive ability of the model for the prognosis of BRCA patients and TNBC patients.

In addition, we compared the immune infiltration status between high- and low-risk groups by different platforms, and different methods verified the same results that the low-risk group had higher immune infiltration, including immune-related cells, immune-related pathways, immune checkpoint expression levels and TMB levels.

Immune checkpoint blockade is an emerging therapeutic strategy that restores the body's antitumour immune response, which is achieved mainly by suppressing the negative regulators of effector T cells and ultimately eradicating cancer cells.

The most common biomarkers of immune checkpoint inhibitors, PD-1, CTLA-4, and CD28 receptors, are present on activated effector T cells that interact with members of their ligand B7 family: B7-1 (CD80), B7-2 (CD-86), or CD274 (PD-L1)^31^. We can use this feature to stratify BRCA patients receiving immunotherapy. In this study, the above immune checkpoints were all highly expressed in the low-risk group, which to some extent suggests that patients in the low-risk group may be more sensitive to immunotherapy and may further prolong the survival of patients in this group.

TMB is the mutational count within the coding region of the tumor genome, expressed as the number of nonsynonymous mutations per megabase (Mut/Mb). Highly mutated neoplasms can generate many neo-antigens that may increase T-cell reactivity. Clinical trials have shown that patients with solid tumors with high TMB (≥ 10 mut/Mb) are more sensitive to immunotherapy and have a better ORR. However, few BRCA patients have high TMB, and only approximately 10% of patients with metastatic BRCA have high TMB and could potentially benefit from immunotherapy^32^.

However, our study has some shortcomings for improvement. First, our study was based on the analysis of existing datasets; therefore, it is necessary to further validate the model in a large cohort. In addition, due to the lack of subtype data, Luminal and HER-2 subtype breast cancers were only sought in the TCGA cohort, but due to limited available subtype data in TCGA, our study was unable to fully analyze these two subtypes.

## Conclusion

In summary, our research had constructed a new BRCA risk scores model based on TANRG. The model showed good predictive performance in both the TCGA training cohort and the GEO validation cohort, and could predict not only the prognosis of BRCA patients but also TME and drug sensitivity, etc. In addition, BRCA patients in the high-risk group have better immune infiltration and immune checkpoint expression, so the model can also guide chemotherapy and immunotherapy for BRCA patients.

### Supplementary Information

Supplementary Figure S1.

Supplementary Figure S2.

Supplementary Table S1.

Supplementary Table S2.

Supplementary Table S3.

## Data Availability

All samples and files were supported by the GEO (https://www.ncbi.nlm.nih.gov/geo) and TCGA databases (http://www.cancer.gov/tcga).

## References

[1] Giaquinto AN, Sung H, Miller KD, Kramer JL, Newman LA, Minihan A (2022). Breast cancer statistics, 2022. CA Cancer J. Clin..

[2] Singh DD, Verma R, Tripathi SK, Sahu R, Trivedi P, Yadav DK (2021). Breast cancer transcriptional regulatory network reprogramming by using the CRISPR/Cas9 system: An oncogenetics perspective. Curr. Top. Med. Chem..

[3] Luo Y, Ye Y, Chen Y, Zhang C, Sun Y, Wang C (2023). A degradome-based prognostic signature that correlates with immune infiltration and tumor mutation burden in breast cancer. Front. Immunol..

[4] Chen M, Wu W, Wang S, Lai X, Liu M, Sun Y (2022). Neutrophils as emerging immunotherapeutic targets: Indirect treatment of tumors by regulating the tumor immune environment based on a sialic acid derivative-modified nanocomplex platform. Int. J. Pharm..

[5] Timaxian C, Vogel CFA, Orcel C, Vetter D, Durochat C, Chinal C (2021). Pivotal role for Cxcr2 in regulating tumor-associated neutrophil in breast cancer. Cancers (Basel).

[6] Zhang Y, Guoqiang L, Sun M, Lu X (2020). Targeting and exploitation of tumor-associated neutrophils to enhance immunotherapy and drug delivery for cancer treatment. Cancer Biol. Med..

[7] Bodac A, Meylan E (2021). Neutrophil metabolism in the cancer context. Semin. Immunol..

[8] Shaul ME, Fridlender ZG (2019). Tumour-associated neutrophils in patients with cancer. Nat. Rev. Clin. Oncol..

[9] Peng B, Lou H, Chen C, Wang L, Li H, Lu T (2022). Mitochondrial homeostasis-related lncRNAs are potential biomarkers for predicting prognosis and immune response in lung adenocarcinoma. Front. Genet..

[10] Yang J, Wang C, Zhang Y, Cheng S, Wu M, Gu S (2023). A novel autophagy-related gene signature associated with prognosis and immune microenvironment in ovarian cancer. J. Ovarian Res..

[11] Goeman JJ (2010). L1 penalized estimation in the Cox proportional hazards model. Biom. J..

[12] Hanzelmann S, Castelo R, Guinney J (2013). GSVA: Gene set variation analysis for microarray and RNA-seq data. BMC Bioinform..

[13] Shen S, Wang G, Zhang R, Zhao Y, Yu H, Wei Y (2019). Development and validation of an immune gene-set based Prognostic signature in ovarian cancer. EBioMedicine.

[14] Ma B, Li Y, Ren Y (2020). Identification of a 6-lncRNA prognostic signature based on microarray re-annotation in gastric cancer. Cancer Med..

[15] Yoshihara K, Shahmoradgoli M, Martinez E, Vegesna R, Kim H, Torres-Garcia W (2013). Inferring tumour purity and stromal and immune cell admixture from expression data. Nat. Commun..

[16] Shankavaram UT, Reinhold WC, Nishizuka S, Major S, Morita D, Chary KK (2007). Transcript and protein expression profiles of the NCI-60 cancer cell panel: An integromic microarray study. Mol. Cancer Ther..

[17] Shankavaram UT, Varma S, Kane D, Sunshine M, Chary KK, Reinhold WC (2009). Cell miner: A relational database and query tool for the NCI-60 cancer cell lines. BMC Genom..

[18] Kono K, Nakajima S, Mimura K (2020). Current status of immune checkpoint inhibitors for gastric cancer. Gastric Cancer.

[19] Antuamwine BB, Bosnjakovic R, Hofmann-Vega F, Wang X, Theodosiou T, Iliopoulos I (2022). N1 versus N2 and PMN-MDSC: A critical appraisal of current concepts on tumor-associated neutrophils and new directions for human oncology. Immunol. Rev..

[20] Qiu J, Xu L, Zeng X, Wu H, Liang F, Lv Q (2022). CCL5 mediates breast cancer metastasis and prognosis through CCR5/Treg cells. Front. Oncol..

[21] Rizeq B, Malki MI (2020). The role of CCL21/CCR7 chemokine axis in breast cancer progression. Cancers (Basel).

[22] Wu L, Sun S, Qu F, Sun M, Liu X, Sun Q (2022). CXCL9 influences the tumor immune microenvironment by stimulating JAK/STAT pathway in triple-negative breast cancer. Cancer Immunol. Immunother..

[23] Zhang R, Zhang S, Xing R, Zhang Q (2019). High expression of EZR (ezrin) gene is correlated with the poor overall survival of breast cancer patients. Thorac. Cancer.

[24] Chen R, Wang X, Fu J, Liang M, Xia T (2022). High FLT3 expression indicates favorable prognosis and correlates with clinicopathological parameters and immune infiltration in breast cancer. Front. Genet..

[25] Aljohani AI, Toss MS, Kurozumi S, Joseph C, Aleskandarany MA, Miligy IM (2020). The prognostic significance of wild-type isocitrate dehydrogenase 2 (IDH2) in breast cancer. Breast Cancer Res. Treat..

[26] Liou AK, Soon G, Tan L, Peng Y, Cher BM, Goh BC (2020). Elevated IL18 levels in nasopharyngeal carcinoma induced PD-1 expression on NK cells in TILS leading to poor prognosis. Oral Oncol..

[27] Hu H, Sun J, Wang C, Bu X, Liu X, Mao Y (2017). IL-33 facilitates endocrine resistance of breast cancer by inducing cancer stem cell properties. Biochem. Biophys. Res. Commun..

[28] Shani O, Vorobyov T, Monteran L, Lavie D, Cohen N, Raz Y (2020). Fibroblast-derived IL33 facilitates breast cancer metastasis by modifying the immune microenvironment and driving type 2 immunity. Cancer Res..

[29] Xie Y, Liu Y, Fan X, Zhang L, Li Q, Li S (2019). Biosci. Rep..

[30] Jiang H, Li H (2021). Prognostic values of tumoral MMP2 and MMP9 overexpression in breast cancer: A systematic review and meta-analysis. BMC Cancer.

[31] Nicolini A, Ferrari P, Carpi A (2022). Immune checkpoint inhibitors and other immune therapies in breast cancer: A new paradigm for prolonged adjuvant immunotherapy. Biomedicines.

[32] Cejuela M, Vethencourt A, Pernas S (2022). Immune checkpoint inhibitors and novel immunotherapy approaches for breast cancer. Curr. Oncol. Rep..

