# Supplementary figures and images for: A novel tumor-associated neutrophil gene signature for predicting prognosis, tumor immune microenvironment, and therapeutic response in breast cancer
Source: Sci Rep. 2024 Mar 4;14:5339. doi: 10.1038/s41598-024-55513-8 (PMC10912776; doi:10.1038/s41598-024-55513-8)

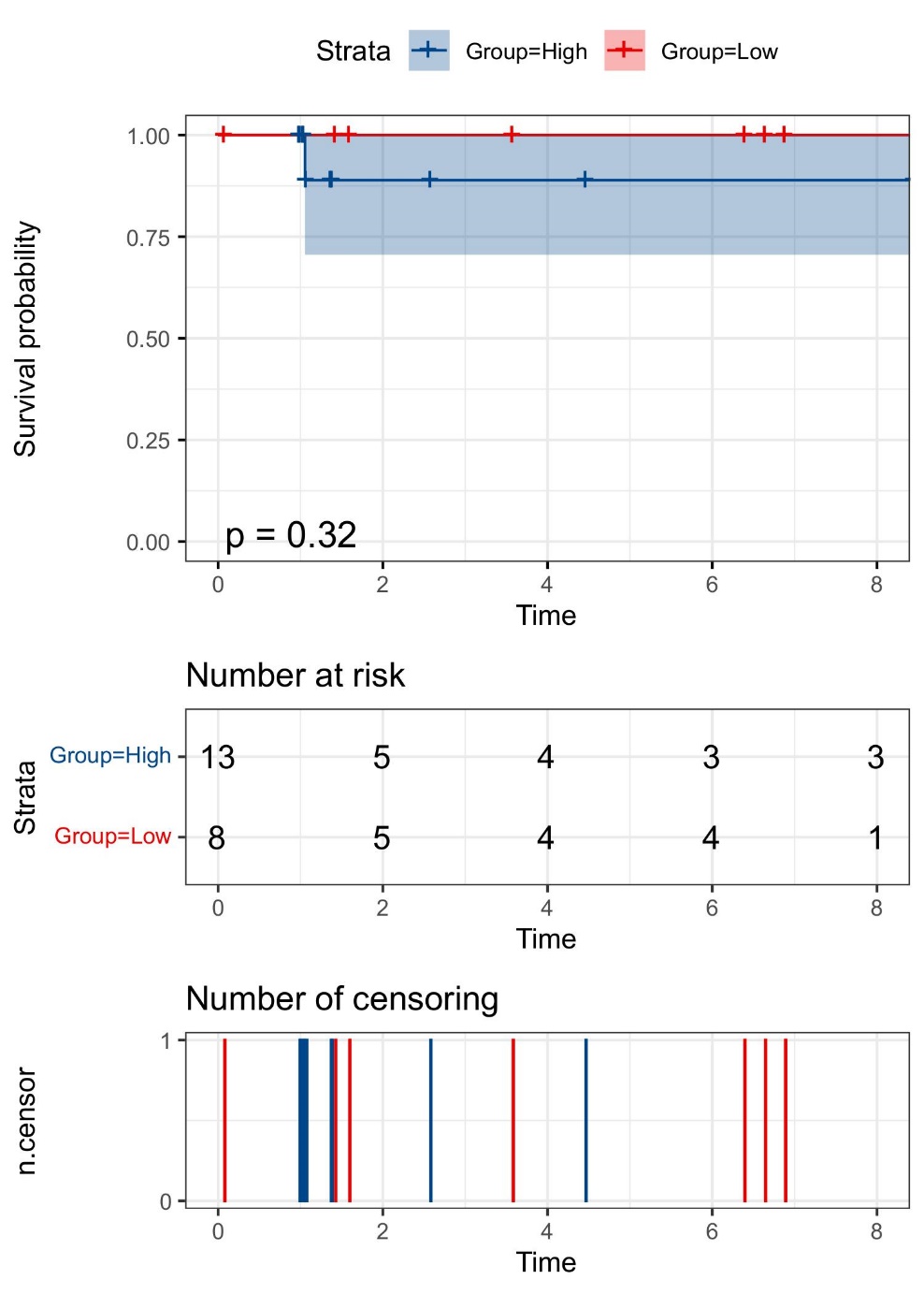


Figure S1

Figure S1. The prognostic performance of the 11 TANRG signature in the Her-2 breast cancer.

Supplement: Supplementary file 1 — Supplementary Figure S1. [file 41598_2024_55513_MOESM1_ESM.docx]
