# Supplementary material for: A novel tumor-associated neutrophil gene signature for predicting prognosis, tumor immune microenvironment, and therapeutic response in breast cancer
Source: Sci Rep. 2024 Mar 4;14:5339. doi: 10.1038/s41598-024-55513-8 (PMC10912776; doi:10.1038/s41598-024-55513-8)
Supplement: Supplementary file 2 — Supplementary Figure S2. [file 41598_2024_55513_MOESM2_ESM.docx]

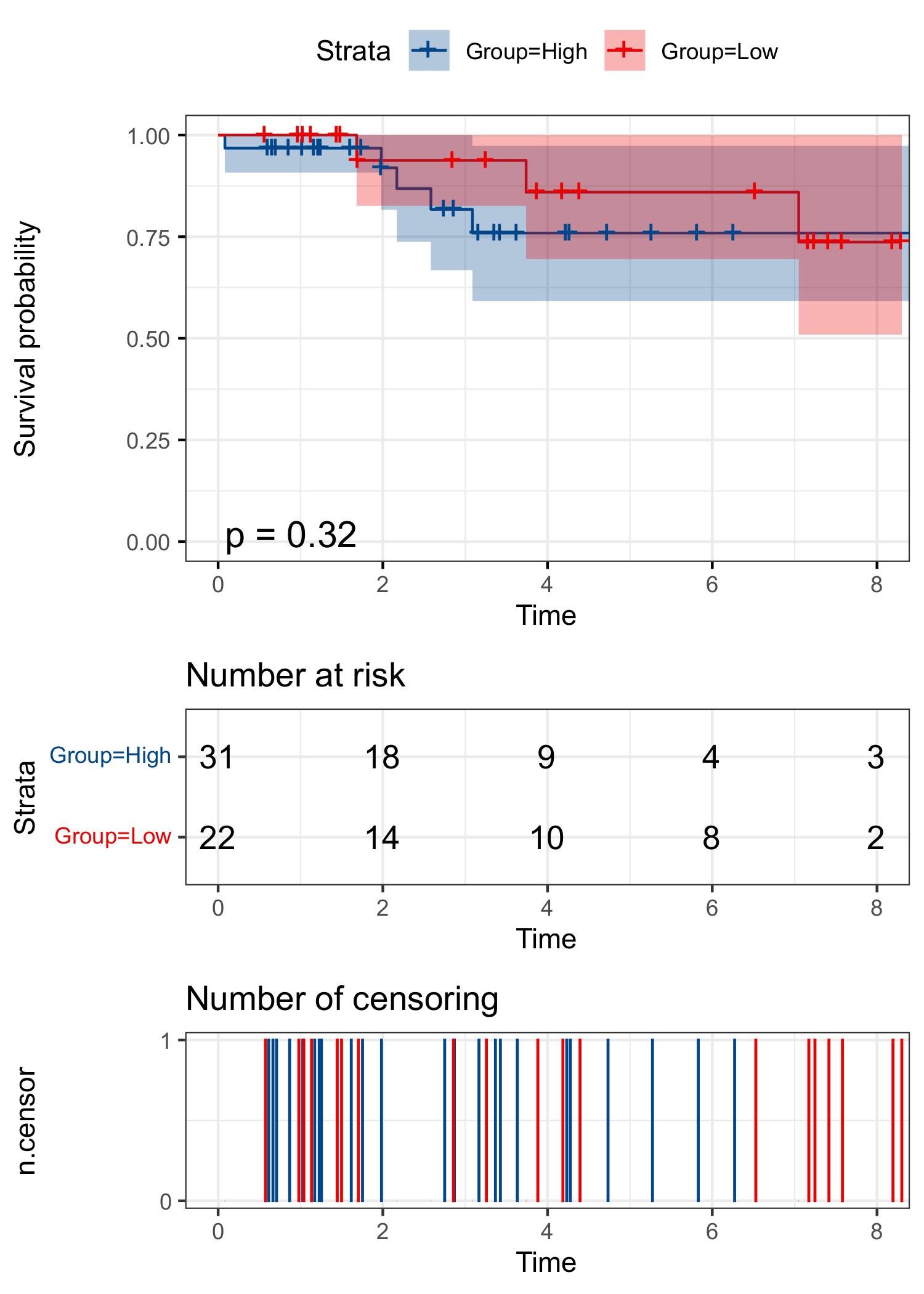


Figure S2

Figure S2. The prognostic performance of the 11 TANRG signature in the Luminal breast cancer.
