# Supplementary material for: A novel tumor-associated neutrophil gene signature for predicting prognosis, tumor immune microenvironment, and therapeutic response in breast cancer
Source: Sci Rep. 2024 Mar 4;14:5339. doi: 10.1038/s41598-024-55513-8 (PMC10912776; doi:10.1038/s41598-024-55513-8)
Supplement: Supplementary file 3 — Supplementary Table S1. [file 41598_2024_55513_MOESM3_ESM.docx]

**Table S1 The 150 differentially expressed genes in BRCA and normal tissues through the “limma” R package with the specific criteria of false discovery rate (FDR) <0.05 and Log_2_ fold change (FC) ≥1 in TCGA cohort.**

| **Gene** | **logFC** | ***P*-value** | **FDR** |
| --- | --- | --- | --- |
| **MUC5AC** | 4.45927898 | 7.51E-06 | 1.22E-05 |
| **SERPINC1** | 2.34725979 | 9.49E-13 | 2.18E-12 |
| **TERT** | 2.60519096 | 1.33E-18 | 3.99E-18 |
| **MMP8** | 1.28879833 | 0.0005911 | 0.00085233 |
| **ADORA2A** | 1.07731916 | 0.00395029 | 0.00530264 |
| **IL17F** | 2.20100362 | 0.01605684 | 0.02039036 |
| **CD80** | 2.21492382 | 1.68E-32 | 1.07E-31 |
| **CEBPE** | 1.1011432 | 3.47E-14 | 8.56E-14 |
| **CCL20** | 2.38911911 | 3.43E-12 | 7.71E-12 |
| **CCL11** | 4.08003192 | 2.10E-46 | 4.27E-45 |
| **TTN** | -3.6650223 | 4.13E-22 | 1.55E-21 |
| **CCR3** | 1.26123407 | 5.16E-10 | 1.04E-09 |
| **CXCL11** | 3.63136378 | 3.54E-33 | 2.40E-32 |
| **CA9** | 4.65573973 | 0.00293009 | 0.00396893 |
| **CCR4** | 1.6130115 | 2.71E-18 | 7.93E-18 |
| **CAMP** | 4.28827981 | 4.88E-30 | 2.66E-29 |
| **IL13** | -1.209465 | 2.74E-08 | 5.00E-08 |
| **EPO** | 3.67003098 | 2.47E-08 | 4.55E-08 |
| **FUT7** | 1.98559981 | 1.51E-21 | 5.35E-21 |
| **HP** | 1.29767329 | 2.74E-09 | 5.32E-09 |
| **VIP** | -1.8561498 | 4.40E-32 | 2.77E-31 |
| **CTLA4** | 1.20497177 | 9.76E-07 | 1.67E-06 |
| **MAPK10** | -1.7950697 | 4.28E-29 | 2.15E-28 |
| **CCL16** | -2.0585532 | 7.23E-41 | 1.04E-39 |
| **MUC5B** | 2.69866192 | 0.00197182 | 0.0026872 |
| **PDCD1** | 1.72542197 | 1.79E-11 | 3.91E-11 |
| **RETN** | 1.75309347 | 1.17E-05 | 1.86E-05 |
| **NOD2** | 1.36633517 | 1.39E-19 | 4.42E-19 |
| **CXCR2** | -1.9423793 | 4.32E-37 | 3.71E-36 |
| **BIRC5** | 3.52478428 | 3.90E-49 | 1.25E-47 |
| **CCL17** | 1.81480854 | 2.09E-08 | 3.88E-08 |
| **FCGR1A** | 1.60105706 | 2.15E-30 | 1.20E-29 |
| **RPS10** | 1.06201434 | 1.30E-17 | 3.68E-17 |
| **HGF** | -1.0110916 | 2.39E-25 | 1.03E-24 |
| **FLT3** | 1.23646672 | 0.0126302 | 0.01636434 |
| **ANGPT1** | -2.6808737 | 1.46E-44 | 2.71E-43 |
| **FANCD2** | 1.40599099 | 4.41E-35 | 3.52E-34 |
| **CEACAM6** | 4.01107216 | 2.95E-15 | 7.67E-15 |
| **CLEC7A** | 1.23772098 | 2.63E-11 | 5.61E-11 |
| **TREM1** | 1.27113715 | 7.61E-12 | 1.68E-11 |
| **FOXP3** | 2.08741269 | 4.73E-35 | 3.71E-34 |
| **OSM** | 1.53706309 | 1.49E-15 | 3.94E-15 |
| **SPINK1** | -1.9161863 | 0.0413675 | 0.04965172 |
| **CDKN2A** | 2.68599941 | 1.78E-26 | 8.02E-26 |
| **CDKN3** | 2.83110577 | 8.29E-52 | 7.41E-50 |
| **CCR7** | 1.59030478 | 3.51E-08 | 6.36E-08 |
| **CXCL9** | 3.03740946 | 1.60E-19 | 5.03E-19 |
| **CKAP2** | 1.36410089 | 2.63E-37 | 2.50E-36 |
| **CXCL3** | -1.8876641 | 3.17E-37 | 2.95E-36 |
| **LEF1** | 1.96255514 | 4.45E-31 | 2.59E-30 |
| **IL2RB** | 1.02192805 | 0.02105759 | 0.02629258 |
| **GNAI1** | -2.3721267 | 1.60E-48 | 4.48E-47 |
| **ITGAL** | 1.00912448 | 1.82E-11 | 3.95E-11 |
| **PPARA** | -1.3240617 | 1.12E-40 | 1.47E-39 |
| **PRKCA** | -1.0671274 | 1.74E-36 | 1.47E-35 |
| **CXCL10** | 3.46011527 | 3.74E-34 | 2.79E-33 |
| **MMP3** | 2.17694671 | 1.04E-21 | 3.75E-21 |
| **LRRC56** | 1.61340401 | 8.01E-23 | 3.23E-22 |
| **TF** | -1.9744006 | 3.62E-44 | 6.47E-43 |
| **LMNB1** | 2.3258887 | 2.45E-47 | 6.09E-46 |
| **BMP6** | -1.7605404 | 2.00E-42 | 3.08E-41 |
| **IL18** | 1.19447352 | 2.40E-17 | 6.71E-17 |
| **SELL** | 1.56093323 | 5.00E-09 | 9.55E-09 |
| **EGLN3** | 1.57613819 | 1.07E-14 | 2.67E-14 |
| **TLR4** | -1.3731635 | 3.98E-36 | 3.29E-35 |
| **IL2RG** | 1.18590155 | 4.30E-05 | 6.67E-05 |
| **CSF3R** | 1.05729731 | 5.68E-05 | 8.70E-05 |
| **FGF2** | -2.5710573 | 5.57E-49 | 1.66E-47 |
| **ERG** | -1.5799081 | 7.46E-48 | 1.96E-46 |
| **MMP9** | 3.05334539 | 2.20E-25 | 9.53E-25 |
| **ST3GAL4** | 1.05185925 | 2.04E-26 | 9.11E-26 |
| **PLA2G4A** | -1.448875 | 4.99E-44 | 8.59E-43 |
| **CD209** | -2.4319792 | 2.01E-33 | 1.40E-32 |
| **KDR** | -1.0463637 | 5.91E-33 | 3.94E-32 |
| **DES** | -3.9162397 | 1.25E-33 | 8.87E-33 |
| **EGF** | -1.4034262 | 1.44E-12 | 3.27E-12 |
| **TFRC** | 1.11048608 | 9.64E-15 | 2.42E-14 |
| **PLAUR** | 1.72310374 | 1.65E-35 | 1.34E-34 |
| **PTGS2** | -1.6504502 | 7.45E-35 | 5.74E-34 |
| **SELP** | -1.6344241 | 7.72E-35 | 5.85E-34 |
| **PTX3** | -1.1608658 | 2.87E-31 | 1.73E-30 |
| **CSF3** | -3.6322888 | 2.98E-29 | 1.51E-28 |
| **ESR1** | 1.64346529 | 9.65E-08 | 1.72E-07 |
| **C1orf35** | 1.19120639 | 2.83E-33 | 1.95E-32 |
| **STAT1** | 1.18537916 | 5.41E-17 | 1.49E-16 |
| **MET** | -1.6293391 | 6.22E-43 | 9.93E-42 |
| **SELE** | -1.2654612 | 0.00011009 | 0.00016514 |
| **NR3C1** | -1.6009791 | 2.00E-50 | 9.92E-49 |
| **EGFR** | -1.7716676 | 6.48E-50 | 2.41E-48 |
| **FOXO1** | -1.72309 | 1.95E-51 | 1.46E-49 |
| **PDGFRA** | -1.3131847 | 1.14E-39 | 1.24E-38 |
| **MME** | -3.2211325 | 1.48E-54 | 6.63E-52 |
| **S100A8** | 3.40248637 | 0.02062073 | 0.02589175 |
| **IL33** | -2.6463068 | 3.11E-52 | 6.95E-50 |
| **ANXA3** | -1.2917477 | 4.87E-34 | 3.57E-33 |
| **PPARG** | -3.2094515 | 4.52E-47 | 1.06E-45 |
| **CTSG** | -1.6863324 | 8.92E-26 | 3.91E-25 |
| **SPP1** | 2.3344332 | 2.26E-24 | 9.44E-24 |
| **PLAU** | 1.38500273 | 5.34E-17 | 1.48E-16 |
| **FCGR3A** | 1.22119653 | 2.65E-18 | 7.79E-18 |
| **EDN1** | -1.3201916 | 2.01E-29 | 1.03E-28 |
| **THBD** | -1.0667018 | 5.35E-24 | 2.21E-23 |
| **RARA** | 1.27776767 | 1.47E-21 | 5.27E-21 |
| **FN1** | 2.90576809 | 3.90E-43 | 6.46E-42 |
| **CA12** | 1.65755153 | 1.26E-13 | 2.99E-13 |
| **F3** | -1.6760721 | 1.11E-40 | 1.47E-39 |
| **BAX** | 1.13697042 | 8.82E-38 | 8.76E-37 |
| **SERPINE1** | 1.03524928 | 2.67E-10 | 5.45E-10 |
| **BCL6** | -1.0417728 | 1.01E-39 | 1.13E-38 |
| **STAT5B** | -1.1234002 | 3.28E-50 | 1.33E-48 |
| **IL6** | -2.5994174 | 2.31E-26 | 1.02E-25 |
| **PIK3R1** | -1.3676079 | 1.85E-38 | 1.97E-37 |
| **PSMD3** | 1.42948236 | 1.26E-32 | 8.27E-32 |
| **CCND1** | 1.27460166 | 1.38E-08 | 2.57E-08 |
| **CCL5** | 1.00389857 | 0.01909806 | 0.02411535 |
| **STAT5A** | -1.3579855 | 2.31E-42 | 3.44E-41 |
| **S100B** | -2.7458856 | 9.15E-47 | 1.95E-45 |
| **ISG15** | 3.43570728 | 3.31E-37 | 3.02E-36 |
| **MUC1** | 2.41365875 | 3.75E-27 | 1.76E-26 |
| **CD34** | -2.0006803 | 3.19E-50 | 1.33E-48 |
| **CXCR4** | 1.10805887 | 2.01E-19 | 6.29E-19 |
| **ERBB2** | 1.74336505 | 1.28E-10 | 2.67E-10 |
| **ACKR3** | -1.6901709 | 6.18E-22 | 2.26E-21 |
| **SIRPA** | -1.0993266 | 9.21E-40 | 1.06E-38 |
| **VWF** | -1.9181773 | 2.04E-40 | 2.53E-39 |
| **CAT** | -1.871059 | 1.78E-50 | 9.92E-49 |
| **PECAM1** | -1.5332373 | 4.68E-38 | 4.87E-37 |
| **CXCL2** | -3.3342037 | 7.88E-51 | 5.03E-49 |
| **KIT** | -2.9858312 | 1.58E-37 | 1.54E-36 |
| **S100A9** | 2.77730505 | 0.01816187 | 0.02299818 |
| **CD36** | -3.9748308 | 1.31E-49 | 4.52E-48 |
| **EMP1** | -1.7849386 | 7.64E-40 | 8.99E-39 |
| **IDH2** | 1.27939561 | 1.61E-32 | 1.04E-31 |
| **EZR** | 1.23181593 | 3.96E-37 | 3.47E-36 |
| **LEP** | -5.818531 | 6.63E-47 | 1.48E-45 |
| **CXCL12** | -1.8453561 | 4.80E-46 | 9.34E-45 |
| **CTSD** | 1.37061096 | 9.81E-25 | 4.14E-24 |
| **MDK** | 1.35965785 | 4.51E-15 | 1.16E-14 |
| **ACKR1** | -2.1495 | 2.04E-40 | 2.53E-39 |
| **EPCAM** | 1.27844656 | 2.92E-27 | 1.40E-26 |
| **SOCS3** | -1.6147369 | 5.03E-14 | 1.24E-13 |
| **CX3CL1** | -2.3729409 | 8.26E-41 | 1.15E-39 |
| **ANXA1** | -2.2740533 | 7.27E-52 | 7.41E-50 |
| **CCL21** | -2.2659858 | 9.40E-34 | 6.78E-33 |
| **CALR** | 1.00190135 | 6.37E-38 | 6.47E-37 |
| **TIMP1** | 1.134324 | 2.93E-22 | 1.12E-21 |
| **HSPB1** | 1.61856408 | 4.37E-22 | 1.63E-21 |
| **JUN** | -1.308615 | 2.09E-31 | 1.28E-30 |
| **VIM** | -1.3380592 | 1.63E-31 | 1.01E-30 |
| **GSN** | -2.4107048 | 4.93E-52 | 7.35E-50 |
