# Supplementary material for: A novel tumor-associated neutrophil gene signature for predicting prognosis, tumor immune microenvironment, and therapeutic response in breast cancer
Source: Sci Rep. 2024 Mar 4;14:5339. doi: 10.1038/s41598-024-55513-8 (PMC10912776; doi:10.1038/s41598-024-55513-8)
Supplement: Supplementary file 4 — Supplementary Table S2. [file 41598_2024_55513_MOESM4_ESM.docx]

**Table S2 The profile of significantly differently infiltrated immune cells between risk groups in different platforms.**

| **Immune cells** | **Platform** | ***P*-value** |
| --- | --- | --- |
| B cell | TIMER | 1.43E-05 |
| T cell CD4+ | TIMER | 2.04E-30 |
| T cell CD8+ | TIMER | 1.65E-29 |
| Neutrophil | TIMER | 2.22E-29 |
| Macrophage | TIMER | 0.00061398 |
| Myeloid dendritic cell | TIMER | 7.78E-28 |
| B cell memory | CIBERSORT | 0.00469915 |
| T cell CD8+ | CIBERSORT | 5.57E-22 |
| T cell CD4+ memory resting | CIBERSORT | 4.73E-09 |
| T cell CD4+ memory activated | CIBERSORT | 3.02E-09 |
| T cell follicular helper | CIBERSORT | 0.00318145 |
| T cell gamma delta | CIBERSORT | 4.75E-08 |
| NK cell resting | CIBERSORT | 2.10E-06 |
| NK cell activated | CIBERSORT | 2.73E-05 |
| Monocyte | CIBERSORT | 0.04554382 |
| Macrophage M0 | CIBERSORT | 1.28E-08 |
| Macrophage M1 | CIBERSORT | 1.17E-17 |
| Macrophage M2 | CIBERSORT | 1.57E-13 |
| Myeloid dendritic cell resting | CIBERSORT | 2.27E-06 |
| Myeloid dendritic cell activated | CIBERSORT | 0.00978732 |
| Eosinophil | CIBERSORT | 0.04874905 |
| Neutrophil | CIBERSORT | 0.00033081 |
| B cell naive | CIBERSORT-ABS | 1.99E-12 |
| B cell memory | CIBERSORT-ABS | 0.00049157 |
| B cell plasma | CIBERSORT-ABS | 7.11E-07 |
| T cell CD8+ | CIBERSORT-ABS | 1.02E-39 |
| T cell CD4+ memory resting | CIBERSORT-ABS | 1.06E-27 |
| T cell CD4+ memory activated | CIBERSORT-ABS | 1.63E-09 |
| T cell follicular helper | CIBERSORT-ABS | 4.15E-27 |
| T cell regulatory (Tregs) | CIBERSORT-ABS | 0.00036032 |
| T cell gamma delta | CIBERSORT-ABS | 4.49E-10 |
| NK cell resting | CIBERSORT-ABS | 1.14E-05 |
| NK cell activated | CIBERSORT-ABS | 3.80E-17 |
| Monocyte | CIBERSORT-ABS | 1.75E-07 |
| Macrophage M1 | CIBERSORT-ABS | 3.73E-36 |
| Macrophage M2 | CIBERSORT-ABS | 3.94E-15 |
| Myeloid dendritic cell resting | CIBERSORT-ABS | 5.17E-10 |
| Myeloid dendritic cell activated | CIBERSORT-ABS | 0.02055932 |
| Mast cell activated | CIBERSORT-ABS | 1.61E-08 |
| Neutrophil | CIBERSORT-ABS | 0.00178775 |
| B cell | QUANTISEQ | 1.85E-29 |
| Macrophage M1 | QUANTISEQ | 0.00018178 |
| Macrophage M2 | QUANTISEQ | 2.19E-07 |
| Neutrophil | QUANTISEQ | 0.02555912 |
| NK cell | QUANTISEQ | 0.0110686 |
| T cell CD8+ | QUANTISEQ | 1.50E-40 |
| T cell regulatory (Tregs) | QUANTISEQ | 2.42E-20 |
| Myeloid dendritic cell | QUANTISEQ | 0.00092746 |
| uncharacterized cell | QUANTISEQ | 7.87E-27 |
| T cell | MCPCOUNTER | 4.46E-43 |
| T cell CD8+ | MCPCOUNTER | 7.21E-35 |
| cytotoxicity score | MCPCOUNTER | 8.72E-37 |
| NK cell | MCPCOUNTER | 3.47E-40 |
| B cell | MCPCOUNTER | 2.89E-31 |
| Monocyte | MCPCOUNTER | 4.73E-11 |
| Macrophage/Monocyte | MCPCOUNTER | 4.73E-11 |
| Myeloid dendritic cell | MCPCOUNTER | 5.41E-44 |
| Endothelial cell | MCPCOUNTER | 5.33E-15 |
| Cancer associated fibroblast | MCPCOUNTER | 0.0066533 |
| Myeloid dendritic cell activated | XCELL | 2.01E-30 |
| B cell | XCELL | 9.25E-13 |
| T cell CD4+ memory | XCELL | 2.48E-11 |
| T cell CD4+ naive | XCELL | 5.71E-11 |
| T cell CD4+ (non-regulatory) | XCELL | 0.03810321 |
| T cell CD4+ effector memory | XCELL | 4.89E-13 |
| T cell CD8+ naive | XCELL | 1.86E-11 |
| T cell CD8+ | XCELL | 2.65E-31 |
| T cell CD8+ central memory | XCELL | 1.11E-42 |
| T cell CD8+ effector memory | XCELL | 0.02700783 |
| Class-switched memory B cell | XCELL | 3.21E-05 |
| Common myeloid progenitor | XCELL | 0.00408459 |
| Myeloid dendritic cell | XCELL | 6.74E-28 |
| Endothelial cell | XCELL | 1.51E-12 |
| Cancer associated fibroblast | XCELL | 2.15E-09 |
| Granulocyte-monocyte progenitor | XCELL | 0.00012815 |
| Hematopoietic stem cell | XCELL | 9.62E-15 |
| Macrophage | XCELL | 0.00026864 |
| Macrophage M1 | XCELL | 1.44E-05 |
| Macrophage M2 | XCELL | 0.00592386 |
| B cell memory | XCELL | 2.99E-14 |
| Monocyte | XCELL | 7.05E-05 |
| B cell naive | XCELL | 2.79E-07 |
| Plasmacytoid dendritic cell | XCELL | 8.78E-15 |
| B cell plasma | XCELL | 0.00271139 |
| T cell CD4+ Th1 | XCELL | 0.00016124 |
| T cell CD4+ Th2 | XCELL | 0.0157669 |
| immune score | XCELL | 1.93E-19 |
| stroma score | XCELL | 4.33E-14 |
| microenvironment score | XCELL | 2.03E-41 |
| B cell | EPIC | 5.29E-29 |
| T cell CD4+ | EPIC | 0.00120936 |
| T cell CD8+ | EPIC | 1.27E-05 |
| Endothelial cell | EPIC | 1.74E-14 |
| Macrophage | EPIC | 1.85E-12 |
| NK cell | EPIC | 1.45E-06 |
| uncharacterized cell | EPIC | 1.78E-09 |
