# Supplementary material for: A novel tumor-associated neutrophil gene signature for predicting prognosis, tumor immune microenvironment, and therapeutic response in breast cancer
Source: Sci Rep. 2024 Mar 4;14:5339. doi: 10.1038/s41598-024-55513-8 (PMC10912776; doi:10.1038/s41598-024-55513-8)
Supplement: Supplementary file 5 — Supplementary Table S3. [file 41598_2024_55513_MOESM5_ESM.docx]

**Table S3 Sensitivity correlation analysis between the independent prognostic genes and drugs based on the CellMiner Database.**

| **Gene** | **Drug** | **Correlation** | **p-value** |
| --- | --- | --- | --- |
| CCL5 | GSK-2194069 | 0.443191375 | 0.00049377 |
| CCL5 | Cyclobenzaprine Hydrochloride | 0.435853877 | 0.00062662 |
| CCL5 | JNJ-54302833 | 0.425626802 | 0.0008658 |
| CCL5 | BAY-1163877 | 0.425143534 | 0.00087891 |
| CCR7 | Nelarabine | 0.928087968 | 1.07E-25 |
| CCR7 | Methylprednisolone | 0.925816386 | 2.49E-25 |
| CCR7 | Zalcitabine | 0.68997564 | 2.08E-09 |
| CCR7 | Ribavirin | 0.670973954 | 8.24E-09 |
| CCR7 | Fluphenazine | 0.647727795 | 3.89E-08 |
| CCR7 | Sapacitabine | 0.620221174 | 2.08E-07 |
| CCR7 | Dexamethasone Decadron | 0.612490949 | 3.23E-07 |
| CCR7 | ZM-336372 | 0.604309007 | 5.09E-07 |
| CCR7 | Chelerythrine | 0.585802061 | 1.36E-06 |
| CCR7 | ST-3595 | 0.555964071 | 5.88E-06 |
| CCR7 | IDOXURIDINE | 0.533429532 | 1.62E-05 |
| CCR7 | PX-316 | 0.519141158 | 2.97E-05 |
| CCR7 | CNDAC | 0.480048195 | 0.00013712 |
| CCR7 | SNS-314 | 0.469140993 | 0.0002034 |
| CCR7 | Hydroxyurea | 0.455253098 | 0.00032984 |
| CCR7 | FENRETINIDE | 0.450906246 | 0.00038211 |
| CCR7 | Fludarabine | 0.450012305 | 0.00039376 |
| CCR7 | Asparaginase | 0.444402526 | 0.00047448 |
| CCR7 | Pluripotin | -0.423576346 | 0.00092267 |
| CXCL9 | Alectinib | 0.770344854 | 1.55E-12 |
| CXCL9 | LDK-378 | 0.742265996 | 2.59E-11 |
| CXCL9 | rifa | 0.696856988 | 1.23E-09 |
| CXCL9 | PF-06463922 | 0.633439985 | 9.48E-08 |
| CXCL9 | CEP-14083 | 0.62588807 | 1.49E-07 |
| CXCL9 | Estramustine | 0.617932703 | 2.37E-07 |
| CXCL9 | ensartinib | 0.591627488 | 1.00E-06 |
| CXCL9 | ASP-3026 | 0.581710671 | 1.68E-06 |
| CXCL9 | brigatinib | 0.569336995 | 3.10E-06 |
| CXCL9 | CCT-251545 | 0.567438612 | 3.40E-06 |
| CXCL9 | Carmustine | 0.539802677 | 1.23E-05 |
| CXCL9 | TAE-684 | 0.53166757 | 1.75E-05 |
| CXCL9 | CEP-37440 | 0.530350404 | 1.85E-05 |
| CXCL9 | gilteritinib | 0.518853101 | 3.01E-05 |
| CXCL9 | NMS-E628 | 0.517639181 | 3.17E-05 |
| CXCL9 | Denileukin Diftitox Ontak | 0.50984947 | 4.35E-05 |
| CXCL9 | ciclosporin | 0.50156839 | 6.05E-05 |
| CXCL9 | Crizotinib | 0.484096716 | 0.00011804 |
| CXCL9 | CEP-28122 | 0.46343946 | 0.00024867 |
| CXCL9 | CT-GSK183 | 0.432332171 | 0.00070122 |
| CXCL9 | BMN-673 | 0.430579524 | 0.00074125 |
| EZR | Vemurafenib | -0.576483536 | 2.18E-06 |
| EZR | PLX-8394 | -0.545922799 | 9.32E-06 |
| EZR | ARQ-680 | -0.526492403 | 2.18E-05 |
| EZR | UNC-0638 | -0.510026768 | 4.32E-05 |
| EZR | Tipifarnib | -0.508572079 | 4.58E-05 |
| EZR | Dabrafenib | -0.507587686 | 4.77E-05 |
| EZR | TAK-632 | -0.488096573 | 0.00010162 |
| EZR | auranofin | -0.484824879 | 0.00011488 |
| EZR | SB-590885 | -0.482106629 | 0.00012709 |
| EZR | S-64315 | -0.479698618 | 0.00013889 |
| EZR | delanzomib | -0.473839708 | 0.0001719 |
| EZR | PLX-4720 | -0.471119892 | 0.00018954 |
| EZR | CYC-065 | -0.455029338 | 0.00033236 |
| EZR | MLN-2480 | -0.454906381 | 0.00033375 |
| EZR | GDC-0994 | -0.452529527 | 0.00036177 |
| EZR | ABT-199 | -0.450085573 | 0.00039279 |
| EZR | E-7090 | -0.448312169 | 0.00041679 |
| EZR | Refametinib | -0.446108641 | 0.00044847 |
| EZR | Bafetinib | -0.445067384 | 0.00046419 |
| EZR | A-1210477 | -0.443410703 | 0.00049023 |
| EZR | BGB-283 | -0.441451853 | 0.00052272 |
| EZR | Bortezomib | -0.441154236 | 0.00052782 |
| EZR | AZD-5991 | -0.44052506 | 0.00053876 |
| EZR | HYPOTHEMYCIN | -0.440309727 | 0.00054255 |
| EZR | AMG-176 | -0.438474739 | 0.00057585 |
| EZR | BIX-01294 | -0.437334741 | 0.00059746 |
| EZR | BMS-387032 | -0.434748786 | 0.00064921 |
| EZR | Encorafenib | -0.433823206 | 0.0006687 |
| EZR | Ixazomib | -0.428078049 | 0.00080198 |
| EZR | Avagacestat | -0.423592846 | 0.0009222 |
| EZR | Sulfatinib | -0.422734885 | 0.00094697 |
| EZR | ONX-0914 | -0.42262278 | 0.00095025 |
| FLT3 | Artemether | 0.747929633 | 1.51E-11 |
| FLT3 | GSK-2194069 | 0.697294906 | 1.19E-09 |
| FLT3 | INK-128 | -0.624020709 | 1.66E-07 |
| FLT3 | Hydroxyurea | 0.594646035 | 8.57E-07 |
| FLT3 | Imexon | 0.575609341 | 2.28E-06 |
| FLT3 | JNJ-54302833 | 0.57224527 | 2.69E-06 |
| FLT3 | Nandrolone phenpropionate | 0.557061888 | 5.58E-06 |
| FLT3 | ABT-199 | 0.544856074 | 9.78E-06 |
| FLT3 | Cyclophosphamide | 0.521783931 | 2.66E-05 |
| FLT3 | Chelerythrine | 0.480136261 | 0.00013667 |
| FLT3 | AZD-5991 | 0.47904564 | 0.00014226 |
| FLT3 | AZD-8055 | -0.474719207 | 0.00016653 |
| FLT3 | AZD-3147 | -0.459903353 | 0.00028119 |
| FLT3 | GDC-0349 | -0.457140022 | 0.00030924 |
| FLT3 | S-63845 | 0.430873433 | 0.0007344 |
| FLT3 | Carboplatin | 0.429991579 | 0.00075514 |
| FLT3 | AZD-2014 | -0.42717781 | 0.00082491 |
| IDH2 | Nelarabine | 0.49307655 | 8.41E-05 |
| IDH2 | Zalcitabine | 0.474655585 | 0.00016691 |
| IDH2 | Methylprednisolone | 0.431275379 | 0.00072512 |
| IDH2 | AM-5992 | 0.427403991 | 0.00081909 |
| IDH2 | Hydroxyurea | 0.422232783 | 0.00096174 |
| IL18 | delanzomib | -0.530988924 | 1.80E-05 |
| IL18 | Ixazomib | -0.525996987 | 2.23E-05 |
| IL18 | ONX-0914 | -0.50500577 | 5.28E-05 |
| IL18 | Bortezomib | -0.504525801 | 5.38E-05 |
| IL18 | Pipamperone | -0.495597625 | 7.63E-05 |
| IL18 | BIX-01294 | -0.491271757 | 9.01E-05 |
| IL18 | A-1210477 | -0.476741745 | 0.00015474 |
| IL18 | UNC-0638 | -0.468288377 | 0.00020966 |
| IL18 | ARQ-680 | -0.466141866 | 0.00022618 |
| IL18 | auranofin | -0.461152984 | 0.00026928 |
| IL18 | AZD-3147 | -0.443363245 | 0.00049099 |
| IL18 | S-64315 | -0.442265521 | 0.00050899 |
| IL18 | Dinaciclib | -0.4400994 | 0.00054628 |
| IL18 | Actinomycin D | -0.437074011 | 0.0006025 |
| IL18 | MG-132 | -0.436613057 | 0.00061151 |
| IL18 | Okadaic acid | -0.43321904 | 0.00068171 |
| IL18 | Noscapine | -0.431880246 | 0.00071135 |
| IL18 | Vemurafenib | -0.431042365 | 0.00073048 |
| IL18 | Ezatiostat | -0.429289624 | 0.00077203 |
| IL18 | A-911 | -0.429186805 | 0.00077453 |
| IL18 | Tandutinib | -0.426632278 | 0.00083909 |
| IL18 | E-7090 | -0.422069101 | 0.0009666 |
| IL2RG | Zalcitabine | 0.838329785 | 2.21E-16 |
| IL2RG | Nelarabine | 0.831278389 | 6.62E-16 |
| IL2RG | Chelerythrine | 0.722127571 | 1.58E-10 |
| IL2RG | Sapacitabine | 0.664336233 | 1.30E-08 |
| IL2RG | Methylprednisolone | 0.662437221 | 1.48E-08 |
| IL2RG | Hydroxyurea | 0.618762991 | 2.26E-07 |
| IL2RG | Asparaginase | 0.611355602 | 3.44E-07 |
| IL2RG | XK-469 | 0.604587759 | 5.01E-07 |
| IL2RG | FENRETINIDE | 0.601921621 | 5.80E-07 |
| IL2RG | Artemether | 0.584380375 | 1.46E-06 |
| IL2RG | Fluphenazine | 0.577035096 | 2.12E-06 |
| IL2RG | Imexon | 0.564611148 | 3.90E-06 |
| IL2RG | Chlorambucil | 0.561916837 | 4.44E-06 |
| IL2RG | Cyclophosphamide | 0.561640514 | 4.50E-06 |
| IL2RG | Bendamustine | 0.559667264 | 4.94E-06 |
| IL2RG | Hydroxychloroquine Sulfate | 0.558704277 | 5.17E-06 |
| IL2RG | S-63845 | 0.525944986 | 2.24E-05 |
| IL2RG | Carmustine | 0.522980015 | 2.53E-05 |
| IL2RG | IDOXURIDINE | 0.521389525 | 2.71E-05 |
| IL2RG | Pipobroman | 0.520248227 | 2.84E-05 |
| IL2RG | Ergosterol | 0.518597057 | 3.04E-05 |
| IL2RG | ST-3595 | 0.517528381 | 3.18E-05 |
| IL2RG | DACARBAZINE | 0.515634659 | 3.44E-05 |
| IL2RG | Uracil mustard | 0.511626161 | 4.05E-05 |
| IL2RG | Melphalan | 0.510959006 | 4.16E-05 |
| IL2RG | Ifosfamide | 0.50997682 | 4.33E-05 |
| IL2RG | entosplenitib | -0.509942804 | 4.33E-05 |
| IL2RG | Arsenic trioxide | 0.505160048 | 5.25E-05 |
| IL2RG | Fenretinide | 0.499679028 | 6.52E-05 |
| IL2RG | INK-128 | -0.49802215 | 6.95E-05 |
| IL2RG | Navitoclax | 0.491013037 | 9.10E-05 |
| IL2RG | AM-5992 | 0.484663037 | 0.00011558 |
| IL2RG | CNDAC | 0.482968537 | 0.0001231 |
| IL2RG | DMAPT | 0.478888767 | 0.00014308 |
| IL2RG | Thiotepa | 0.477460923 | 0.00015074 |
| IL2RG | A-1331852 | 0.477405052 | 0.00015105 |
| IL2RG | Etoposide | 0.475781126 | 0.00016024 |
| IL2RG | Triethylenemelamine | 0.473271932 | 0.00017546 |
| IL2RG | Ribavirin | 0.471610818 | 0.00018624 |
| IL2RG | Fludarabine | 0.470212311 | 0.00019579 |
| IL2RG | Lomustine | 0.464796273 | 0.00023714 |
| IL2RG | Masoprocol | 0.463621394 | 0.0002471 |
| IL2RG | PX-316 | 0.456697363 | 0.00031396 |
| IL2RG | Irofulven | -0.45532708 | 0.00032901 |
| IL2RG | Dexamethasone Decadron | 0.455157186 | 0.00033091 |
| IL2RG | Batracylin | 0.454802358 | 0.00033494 |
| IL2RG | ZM-336372 | 0.440473719 | 0.00053966 |
| IL2RG | Pluripotin | -0.438034472 | 0.00058411 |
| IL2RG | Telatinib | -0.434730376 | 0.0006496 |
| IL2RG | AZD-4320 | 0.434309674 | 0.0006584 |
| IL2RG | AZD-5991 | 0.430774372 | 0.0007367 |
| IL2RG | Cytarabine | 0.430279647 | 0.00074831 |
| IL2RG | ciclosporin | 0.429591425 | 0.00076472 |
| IL2RG | 6-THIOGUANINE | 0.427367926 | 0.00082002 |
| IL33 | Volitinib | 0.680282422 | 4.26E-09 |
| IL33 | PF-04217903 | 0.671550636 | 7.92E-09 |
| IL33 | EMD-1204831 | 0.603684077 | 5.26E-07 |
| IL33 | IDEBENONE | 0.454509097 | 0.00033829 |
| IL33 | Rebimastat | 0.425453692 | 0.00087048 |
| MAPK10 | aloin | 0.735529739 | 4.83E-11 |
| MAPK10 | PD183805 | 0.511000666 | 4.15E-05 |
| MAPK10 | S-222611 | 0.486042161 | 0.00010977 |
| MAPK10 | BMS-599626 | 0.473626212 | 0.00017323 |
| MAPK10 | Poziotinib | 0.458202518 | 0.00029816 |
| MAPK10 | Dacomitinib | 0.449077382 | 0.00040628 |
| MAPK10 | Sapitinib | 0.444936427 | 0.0004662 |
| MAPK10 | EXEL-7647 | 0.437018778 | 0.00060357 |
| MAPK10 | AZD-3759 | 0.432295158 | 0.00070204 |
| MAPK10 | Afatinib | 0.431767638 | 0.00071389 |
| MMP9 | Rebimastat | 0.579773011 | 1.85E-06 |
